# The Small RNA Landscape in Azoospermia: Implications for Male Infertility and Sperm Retrieval—A Preliminary Study

**DOI:** 10.3390/ijms26083537

**Published:** 2025-04-09

**Authors:** Maria-Anna Kyrgiafini, Aris Kaltsas, Alexia Chatziparasidou, Zissis Mamuris

**Affiliations:** 1 Laboratory of Genetics, Comparative and Evolutionary Biology, Department of Biochemistry and Biotechnology, University of Thessaly, Viopolis, Mezourlo, 41500 Larissa, Greece; 2 Third Department of Urology, Attikon University Hospital, School of Medicine, National and Kapodistrian University of Athens, 12462 Athens, Greece; 3 Embryolab IVF Unit, St. 173-175 Ethnikis Antistaseos, Kalamaria, 55134 Thessaloniki, Greece

**Keywords:** azoospermia, microRNA (miRNA), male infertility, TESE, pregnancy outcome

## Abstract

MicroRNAs (miRNAs), a class of small noncoding RNAs, play a crucial role in spermatogenesis. However, their specific expression patterns in azoospermic patients, particularly in relation to sperm presence and pregnancy outcomes, remain underexplored. We performed small RNA sequencing on forty testicular tissue samples from idiopathic azoospermic and cryptozoospermic patients who underwent testicular sperm extraction (TESE). Differentially expressed (DE) miRNAs were identified across groups with high, rare, or no spermatozoa presence, as well as between individuals with successful and unsuccessful pregnancies following assisted reproduction. Functional enrichment analyses were conducted to assess the biological relevance of miRNA alterations. Our findings revealed distinct miRNA expression patterns linked to sperm presence and pregnancy outcomes. Samples with high sperm presence exhibited reduced miRNA expression, while those with impaired spermatogenesis demonstrated upregulated miRNAs associated with cell survival and differentiation pathways. Several regulatory pathways were also disrupted in samples leading to unsuccessful pregnancies, including the estrogen signaling receptor (ESR) pathway, interleukin-4 and interleukin-13 signaling, and transcription networks. This study highlights miRNA-mediated regulatory differences in azoospermic patients, identifying potential biomarkers for sperm retrieval success and fertility outcomes. Future validation and multi-omics approaches are needed to confirm these findings and enhance male infertility diagnostics.

## 1. Introduction

Infertility is a disease of the male or female reproductive system defined by the failure to achieve a pregnancy after 12 months or more of regular unprotected sexual intercourse, according to the World Health Organization (WHO). Male infertility is a complex condition defined by the WHO as “the inability of a male to make a fertile female pregnant after at least one year of regular unprotected sexual intercourse”. It is estimated that male-specific factors are solely responsible for approximately 20% of infertility cases and contribute together with female-specific factors to another 30% to 40% of all infertility cases [1]. Among these, azoospermia, characterized by the absence of sperm in the ejaculate, is the most severe form of male infertility [2], affecting approximately 1% of all men and 10–15% of infertile men [3]. Azoospermia is classified into obstructive (OA) and non-obstructive (NOA) forms, with NOA being the most prevalent and challenging due to its association with testicular dysfunction [4].

Before 1995, azoospermia was synonymous with sterility, as there were no therapeutic options available. The introduction of intracytoplasmic sperm injection (ICSI) in 1992 [5], followed by the first successful use of testicular spermatozoa for conception in 1995 [6], revolutionized the treatment landscape for azoospermic men. Today, assisted reproductive technologies (ART) offer the possibility of biological paternity for many men with azoospermia, provided that viable spermatozoa can be retrieved [7]. Testicular sperm extraction (TESE) is a pivotal procedure in this context, allowing for surgical retrieval of sperm directly from testicular tissue and significantly improving the chances of pregnancy [8]. Over time, advances in TESE have led to the development of variants, including multi-biopsy/conventional TESE, microdissection TESE, and testicular sperm aspiration (TESA) [8,9,10]. Beyond its clinical utility, TESE has facilitated research into the molecular and genetic underpinnings of male infertility, paving the way for more personalized and effective treatments.

However, despite advances in ART, the treatment of azoospermia remains a significant challenge, as TESE is not always successful in retrieving viable sperm [11,12]. Sperm retrieval rates for men undergoing microdissection TESE (micro-TESE) have been reported to reach up to 63%. But, outcomes vary widely, particularly in cases of non-obstructive azoospermia (NOA), where retrieval rates are often low and unpredictable [12,13,14]. The variability in micro-TESE success is influenced by the underlying cause and type of azoospermia, with studies showing substantial differences in outcomes based on these factors [12,13,15]. Moreover, TESE is not without risks. Complications such as hematoma, devascularization, inflammation, and a decline in testosterone levels have been reported, highlighting the invasive nature of the procedure [16]. TESE is also costly, emphasizing the need for accurate preoperative identification of patients with the highest likelihood of successful sperm retrieval [17]. These challenges underscore the importance of developing preoperative predictors of TESE success, ideally non-invasive, to improve patient selection and outcomes [18]. Furthermore, even when viable sperm are retrieved, the subsequent use of in vitro fertilization (IVF) or intracytoplasmic sperm injection (ICSI) does not guarantee successful pregnancy outcomes, as multiple factors, including the etiology of the azoospermia, sperm characteristics, etc., can significantly influence the probability of conception [19,20,21].

Small RNAs (sRNAs), including microRNAs (miRNAs), small interfering RNAs (siRNAs), and piwi-interacting RNAs (piRNAs), are critical regulators of gene expression and play essential roles in various biological processes [22,23]. Emerging evidence suggests that small RNAs, such as miRNAs and piRNAs, play a critical role in the regulation of key processes in spermatogenesis, testicular function, and sperm maturation [24,25,26]. More specifically, these non-coding RNAs influence gene silencing, post-transcriptional regulation, and genome stability, making them indispensable for normal testicular function and male fertility [25,27]. Recent studies have demonstrated that the dysregulation of small RNAs is associated with impaired spermatogenesis and male infertility, particularly in conditions such as non-obstructive azoospermia (NOA) [25,28]. The study of sRNA functionality is a rapidly expanding domain within reproductive biology, despite the distinct roles of each subtype. MiRNAs, for example, have been implicated in the fine-tuning of the molecular pathways involved in germ cell development, spermatogenesis, and apoptosis [29,30] while studies have also explored changes in miRNA expression levels in infertile (or subfertile) men compared to controls and have identified several differentially expressed miRNAs [29,31]. Similarly, piRNAs are essential for transposon silencing and maintaining genomic integrity in germ cells [32]. Therefore, disruption of the piRNA pathway can result in male infertility [32,33]. Furthermore, small RNAs, particularly microRNAs (miRNAs), have emerged as promising biomarkers because of their stability, specificity, and ability to reflect both physiological and pathological states [34], making them ideal candidates for applications in reproductive biology and clinical practice.

However, the specific roles of small RNAs in male reproduction and fertility remain poorly understood. In particular, small RNA expression profiles associated with TESE success and their potential influence on pregnancy outcomes have yet to be fully elucidated. Understanding the role of small RNAs in these contexts could not only improve the prediction of TESE outcomes and provide preliminary data on candidate small RNAs for use as biomarkers but also offer insights into the factors that influence pregnancy success, thus addressing a critical gap in the treatment of azoospermic patients.

Therefore, this study aimed to investigate the small RNA expression profiles in testicular tissue samples from 40 azoospermic patients, classified into five distinct groups based on sperm presence and pregnancy outcomes. By performing small RNA sequencing, we sought to identify the differential expression patterns of small RNAs in these categories and uncover their potential roles in spermatogenesis, TESE success, and pregnancy outcomes. To better understand the biological relevance of differentially expressed miRNAs, target prediction analyses were conducted to identify their potential gene targets. This step is critical to elucidate the downstream regulatory pathways influenced by miRNAs, providing insights into the molecular mechanisms underlying azoospermia and its associated clinical outcomes.

## 2. Results

### 2.1. Patients’ Characteristics

The present study included 40 individuals, classified into five groups based on the presence of spermatozoa in testicular tissue and pregnancy outcomes. The characteristics of the individuals are summarized in Table 1. The mean age of the participants across all groups was 35.3 years (range: 28–45). Statistical analyses indicate no significant differences in baseline factors such as age, alcohol consumption, smoking status, or body mass index (BMI) between the groups (*p*-values > 0.05). This comparability ensures that any observed differences are likely attributable to biological or genetic factors, rather than demographic or lifestyle differences.

### 2.2. Small RNA Sequencing and Data Quality

The sequencing data generated for this study were of high quality and consistent across all analyzed samples. The total read count averaged 22,884,318, with individual sample values ranging from 21,604,874 (HIGHSPZ2) to 23,968,227 (RSPZNP2). Following stringent quality control filtering, including the removal of low-quality reads, adapter sequences, and reads shorter than 18 nucleotides, the clean read count averaged 21,506,604. Clean read counts ranged from 20,179,995 (CRYPTO2) to 22,429,360 (RSPZP1), with retention rates between 91.6% and 95.6% of total reads. The mapping efficiency of clean reads to the human reference genome was similarly robust, averaging 74.9% across all samples. Length distribution analysis revealed that small RNAs (sRNAs) predominantly ranged between 18 and 35 nucleotides, which is consistent with the expected profiles for miRNAs, piRNAs, and other small RNA species typically found in human testicular tissue. Sequencing data, including the mapping rates for each sample, are summarized in Table 2.

### 2.3. Identification of Known and Novel miRNAs

**Figure 1:**
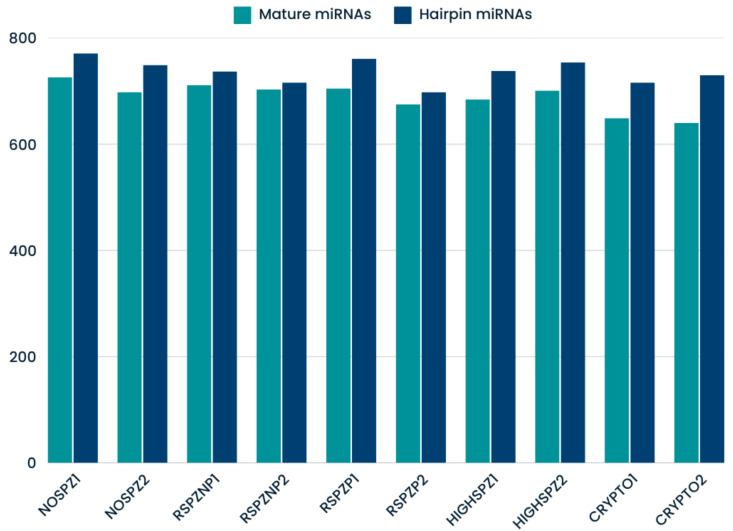
The known mapped miRNAs identified in testes samples.

In this study, a total of 1244 known miRNAs and 84 novel miRNAs were identified across the testis tissue samples, reflecting the transcriptional diversity of the testis. Among the known miRNAs, the number of mapped mature miRNAs ranged from 640 (CRYPTO2) to 726 (NOSPZ1), while the number of mapped hairpin miRNAs ranged from 698 (RSPZP2) to 771 (NOSPZ1), as shown in Figure 1.

**Figure 2:**
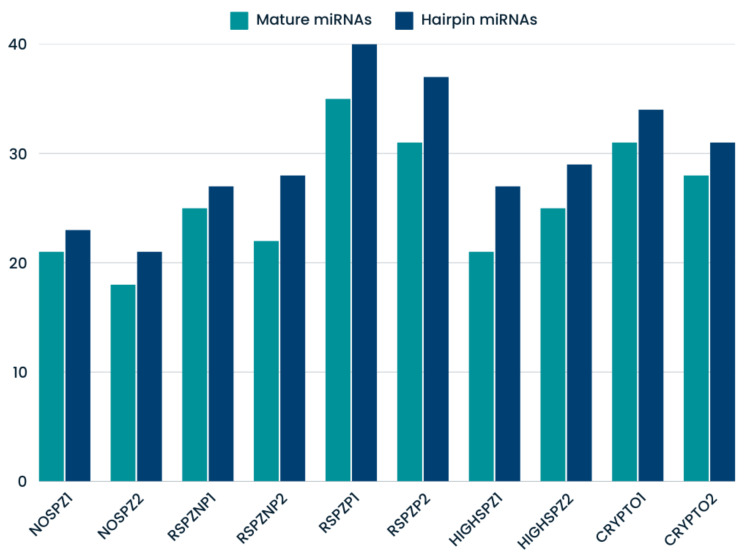
Novel mapped miRNAs identified in corresponding samples.

The number of novel mature miRNAs ranged from 18 (NOSPZ2) to 35 (RSPZP1), while the number of novel hairpin miRNAs ranged from 21 (NOSPZ2) to 40 (RSPZP1) (Figure 2). These findings underscore the complexity of small RNA populations in testis tissue and highlight the potential of novel miRNAs as regulators of spermatogenesis and male infertility.

### 2.4. Differential Expression Profiles of miRNAs

**Figure 3:**
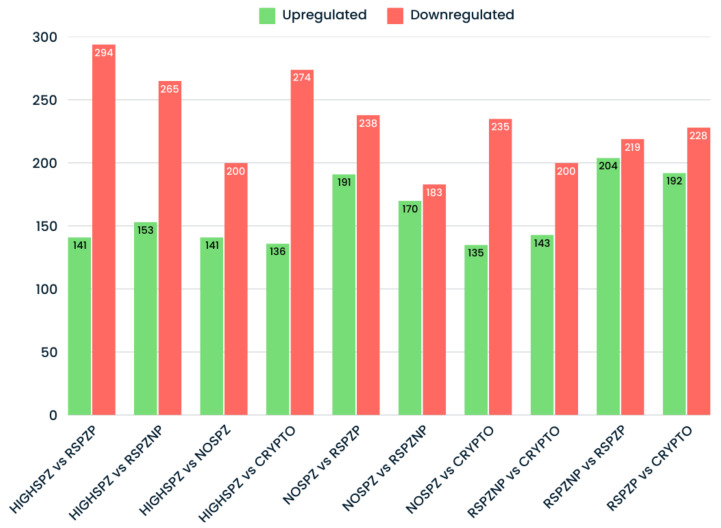
Number of upregulated and downregulated miRNAs identified across pairwise comparisons of studied groups. Upregulated miRNAs are shown in green, while downregulated miRNAs are shown in red. The x-axis represents the pairwise comparisons, while the y-axis represents the number of DE miRNAs.

Analysis of differentially expressed (DE) miRNAs across all group comparisons revealed substantial variability in miRNA expression patterns. A total of 853 unique DE miRNAs were identified, with significant differences observed in the number of upregulated and downregulated miRNAs across the comparisons (Figure 3). Notably, downregulated miRNAs consistently outnumbered upregulated miRNAs in nearly all pairwise comparisons. This trend suggests a global repression of miRNA expression, which may reflect disrupted germ cell activity or dysregulation in miRNA biogenesis pathways. These observations are particularly relevant, given that all samples were derived from patients with azoospermia or cryptozoospermia. Furthermore, comparisons between groups with high spermatozoa presence (HIGHSPZ) and those with reduced spermatozoa presence (NOSPZ, CRYPTO, and RSPZ) consistently showed a predominance of downregulated miRNAs.

**Figure 4:**
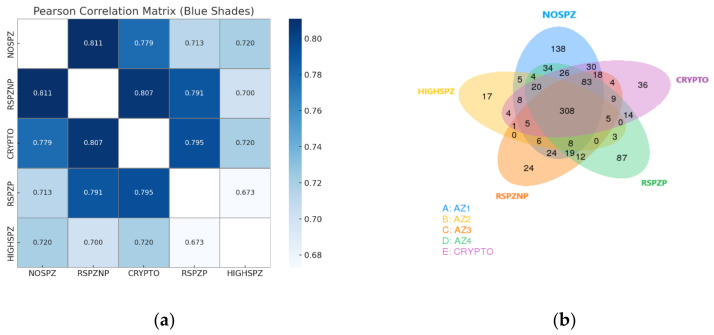
(**a**) Heatmap showing the correlation coefficients of the miRNA expression profiles in all sample groups. Stronger correlations (darker blue) were observed between groups with closer biological relationships, such as NOSPZ and RSPZNP. In contrast, groups with distinct biological characteristics, such as HIGHSPZ and RSPZP, exhibited weaker correlations (lighter blue). (**b**) Venn diagram illustrating the shared and unique miRNAs across the sample groups.

To identify the shared and unique miRNAs across all sample groups, a Venn diagram was generated (Figure 4b). A significant number of miRNAs (308) were found to be shared among all groups, suggesting the involvement of common regulatory pathways in fundamental testicular processes. Additionally, 83 miRNAs were shared exclusively among the CRYPTO, NOSPZ, and RSPZ groups, highlighting the regulatory mechanisms specific to these conditions. Several unique miRNAs were also identified within individual groups, reflecting condition-specific regulatory patterns. Notably, the NOSPZ group exhibited the highest number of group-specific miRNAs (138), potentially indicating distinct molecular adaptations or disruptions uniquely associated with this condition. Furthermore, Spearman’s correlation coefficient analysis of miRNA expression profiles between different sample groups provided insights into their relationships (Figure 4a). Groups with similar biological characteristics exhibited higher correlation coefficients, while more distinct groups demonstrated lower correlations. The highest correlation was observed between the RSPZNP and NOSPZ groups, whereas the lowest correlation was noted between the HIGHSPZ and RSPZP groups, reflecting distinct regulatory landscapes. Overall, the HIGHSPZ group consistently exhibited lower correlation coefficients with all other groups, as expected, due to its biologically distinct profile, which is characterized by robust spermatogenesis.

**Figure 5:**
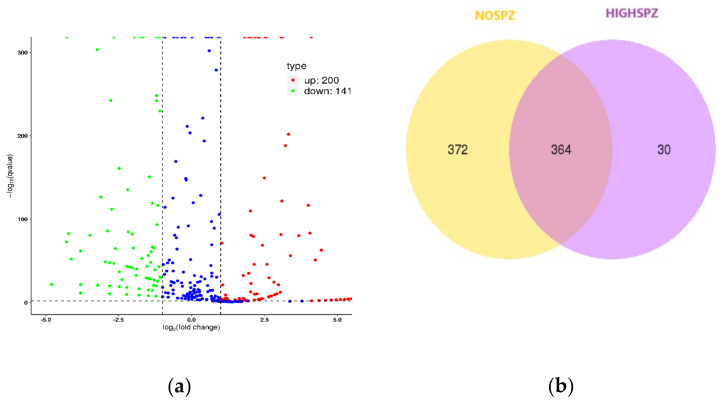
(**a**) Volcano plot illustrating differentially expressed (DE) miRNAs between NOSPZ and HIGHSPZ groups. Significant miRNAs were identified based on adjusted *p*-values (<0.05) and |log_2_ (fold change)| > 1. Upregulated miRNAs are shown in red, and downregulated miRNAs are shown in green. Non-significantly differentially expressed miRNAs are shown in blue. (**b**) Venn diagram depicting the overlap and unique miRNAs between NOSPZ and HIGHSPZ groups.

To gain deeper insights into the role of miRNAs in sperm retrieval and reproductive outcomes, we focused on specific pairwise comparisons. A key comparison was between NOSPZ and HIGHSPZ (NOSPZ vs. HIGHSPZ), representing extreme conditions with a substantial number of differentially expressed (DE) miRNAs and a low correlation of 0.72. Specifically, a volcano plot was generated to visualize the distribution of DE miRNAs, highlighting significant miRNAs with adjusted *p*-values < 0.05 and |log_2_ (fold change)| > 1 (Figure 5a). A total of 341 DE miRNAs were identified, including 200 upregulated and 141 downregulated miRNAs. The top 10 DE miRNAs, showcasing the most pronounced changes in expression, are listed in Table 3. Additionally, a Venn diagram was used to illustrate the overlap and specificity of miRNAs between the NOSPZ and HIGHSPZ groups (Figure 5b). Of the detected miRNAs, 364 were shared between both groups, 372 were unique to NOSPZ, and 30 were exclusive to HIGHSPZ.

This pattern suggests that normal spermatogenesis (HIGHSPZ) may be associated with a global reduction in miRNA expression, while impaired spermatogenesis may involve increased miRNA expression. The latter could reflect compensatory mechanisms or regulatory adaptations in response to decreased sperm production.

**Figure 6:**
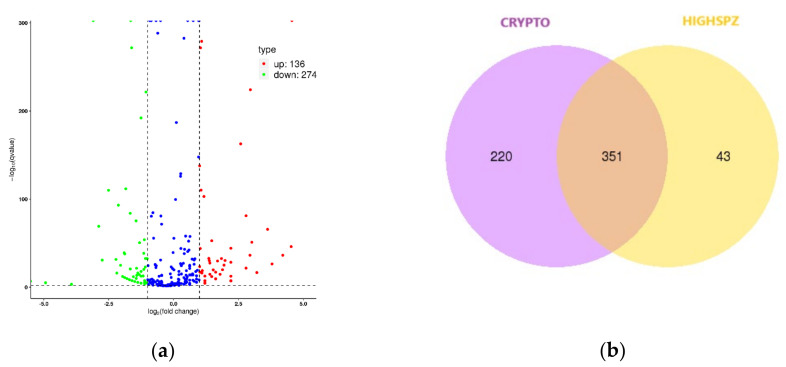
(**a**) Volcano plot illustrating differentially expressed (DE) miRNAs between HIGHSPZ and CRYPTO groups. Significant miRNAs were identified based on adjusted *p*-values (<0.05) and |log_2_ (fold change)| > 1. Upregulated miRNAs are shown in red, and downregulated miRNAs are shown in green. Non-significantly differentially expressed miRNAs are shown in blue. (**b**) Venn diagram depicting the overlap and unique miRNAs between CRYPTO and HIGHSPZ groups.

Similarly, we focused on the comparison between HIGHSPZ and CRYPTO, which, like the HIGHSPZ vs. NOSPZ comparison, contrasts high and low sperm presence. Both comparisons exhibited the same correlation (0.72), and a total of 410 DE miRNAs were identified in the HIGH vs. CRYPTO comparison. As shown in the volcano plot (Figure 6a), 136 miRNAs were upregulated, while 274 were downregulated, reinforcing the trend of predominance of downregulated miRNAs in samples with higher sperm presence. The Venn diagram (Figure 6b) also highlighted overlapping and distinct miRNA expression patterns. Both comparisons revealed a substantial number of shared miRNAs (351 in HIGHSPZ vs. CRYPTO and 364 in HIGHSPZ vs. NOSPZ), while the proportion of group-specific miRNAs was also comparable. HIGHSPZ had 30 unique miRNAs in the CRYPTO comparison versus 43 in the NOSPZ comparison, suggesting that the regulatory divergence between HIGHSPZ and these groups may be similar. This observation highlights the potential involvement of shared pathways or mechanisms contributing to spermatogenic dysfunction in both NOSPZ and CRYPTO. Furthermore, the top 10 DE miRNAs that exhibited the most significant changes in expression are presented in Table 4.

**Figure 7:**
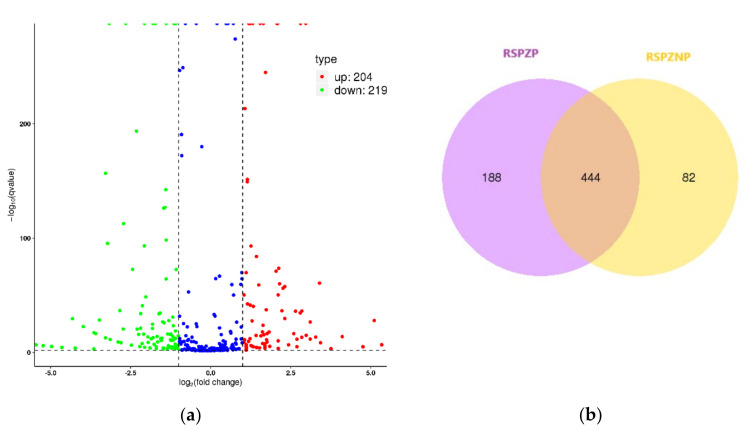
(**a**) Volcano plot illustrating differentially expressed (DE) miRNAs between RSPZNP and RSPZP groups. Significant miRNAs were identified based on adjusted *p*-values (<0.05) and |log_2_ (fold change)| > 1. Upregulated miRNAs are shown in red, and downregulated miRNAs are shown in green. Non-significantly differentially expressed miRNAs are shown in blue. (**b**) Venn diagram depicting the overlap and unique miRNAs between RSPZNP and RSPZP groups.

To further explore the role of miRNAs and their association with reproductive outcomes, we compared the miRNA expression profiles between individuals with rare spermatozoa but no pregnancy (RSPZNP) and those with rare spermatozoa who achieved a successful pregnancy (RSPZP). This pairwise analysis provided valuable insights into both the shared and distinct miRNA regulatory profiles underlying these phenotypes. Notably, a moderate correlation (0.791) in miRNA expression was observed between the groups, suggesting a common core of regulatory elements alongside differential expression patterns that may influence pregnancy outcomes. Differential expression analysis identified a total of 423 miRNAs, with 204 upregulated and 219 downregulated in the RSPZNP group (Figure 7a). Furthermore, a Venn diagram was used to examine the overlap and specificity of miRNA expression between the two groups, revealing 444 miRNAs common to both (Figure 7b). The top 10 differentially expressed miRNAs are listed in Table 5.

### 2.5. Target Prediction of Differentially Expressed miRNAs—GO and KEGG Analyses

To further elucidate the biological functions underlying the observed differences in miRNA expression, we performed a comprehensive target gene analysis of the differentially expressed (DE) miRNAs. To gain deeper insight into the biological processes, cellular components, and molecular functions associated with these target genes, we also conducted Gene Ontology (GO) and KEGG enrichment analyses. These analyses enabled us to identify the key molecular pathways and cellular mechanisms that may contribute to the regulation of spermatogenesis, male reproductive function, and pregnancy outcomes.

**Figure 8:**
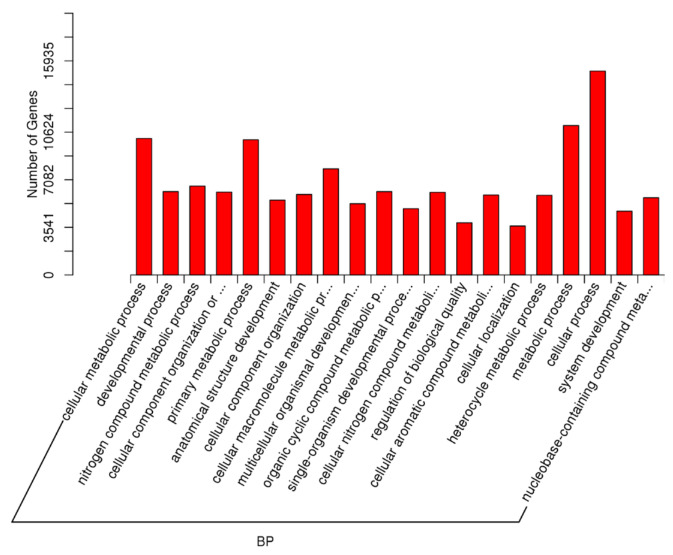
The GO enrichment analysis for the targets of differentially expressed miRNAs (NOSPZ vs. HIGHSPZ) for biological process (BP).

**Figure 9:**
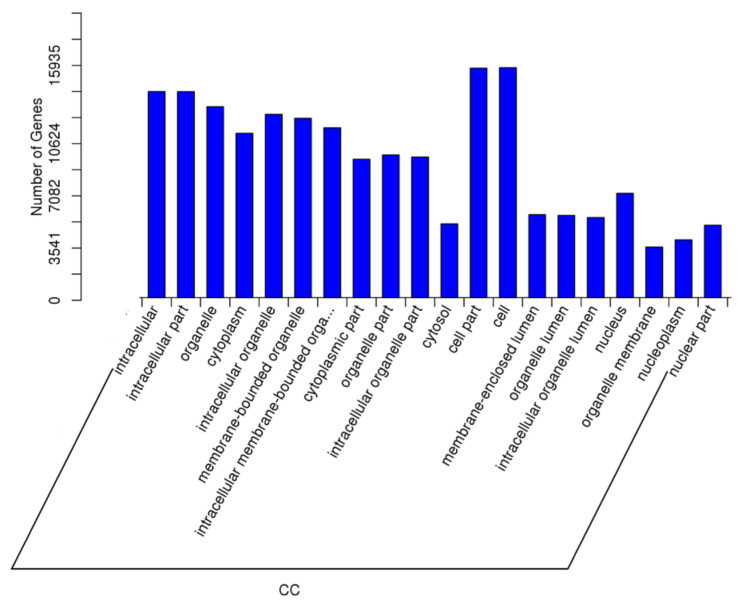
The GO enrichment analysis for the targets of differentially expressed miRNAs (NOSPZ vs. HIGHSPZ) for cellular component (CC).

**Figure 10:**
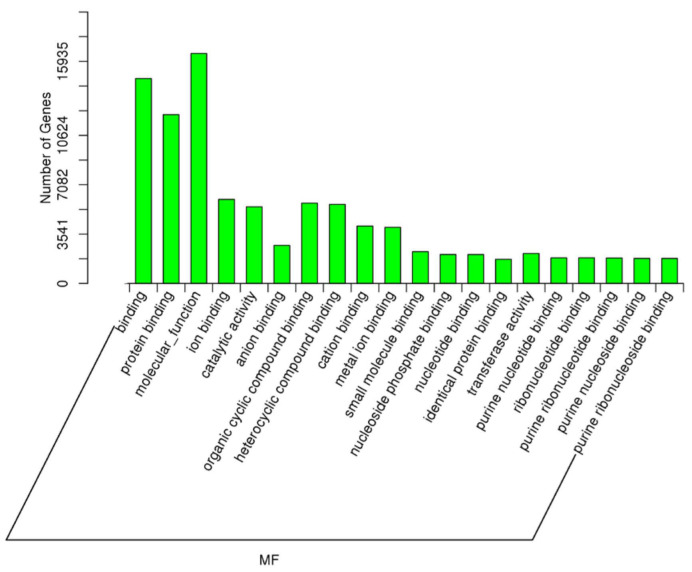
The GO enrichment analysis for the targets of differentially expressed miRNAs (NOSPZ vs. HIGHSPZ) for molecular function (MF).

As described earlier, an important comparison in this study was between the samples with high spermatozoa presence after TESE (HIGHSPZ) and those with no spermatozoa (NOSPZ). For the target genes of DE miRNAs between these samples, regarding GO biological process, the top enriched terms included cellular metabolic process, developmental process, nitrogen compound metabolic process, cellular component organization, primary metabolic process, anatomical structure development, multicellular organism development, and system development. These findings suggest that differentially expressed miRNAs play a crucial role in cellular growth, metabolism, and structural organization, all of which are essential for spermatogenesis. For the GO cellular component, the most enriched categories were intracellular, intracellular part, organelle, cytoplasm, membrane-bounded organelle, nucleus, and nucleoplasm. These results indicate that miRNA-regulated genes are primarily localized within intracellular environments, particularly in nuclear and cytoplasmic compartments, where they may regulate key transcriptional and post-transcriptional processes. Finally, regarding GO molecular function, the most significantly enriched terms included protein binding, molecular function regulation, metal ion binding, catalytic activity, nucleoside phosphate binding, and purine nucleotide binding (Figure 8, Figure 9 and Figure 10).

To further explore the functional impact of DE miRNAs, we conducted pathway enrichment analysis focusing specifically on the gene targets of upregulated and downregulated miRNAs in the NOSPZ vs. HIGHSPZ comparison. The top five enriched pathways are summarized in Table 6.

**Figure 11:**
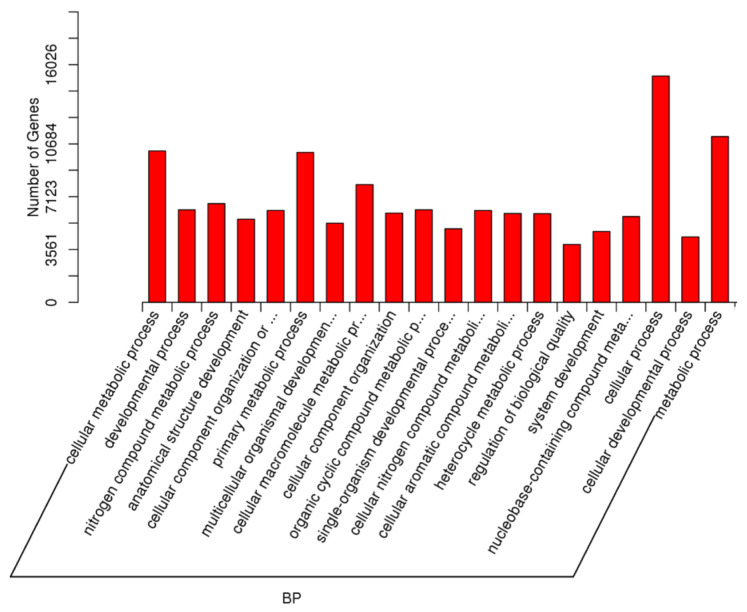
The GO enrichment analysis for the targets of differentially expressed miRNAs (RSPZNP vs. RSPZP) for biological process (BP).

**Figure 12:**
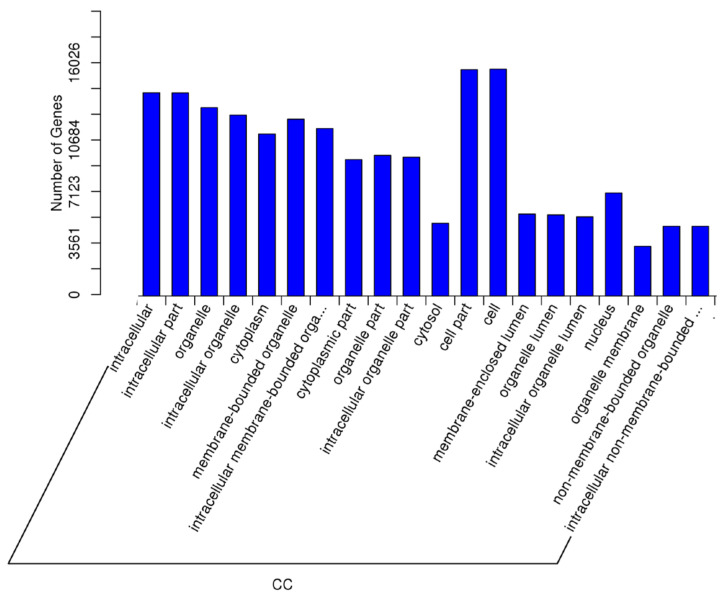
The GO enrichment analysis for the targets of differentially expressed miRNAs (RSPZNP vs. RSPZP) for cellular component (CC).

**Figure 13:**
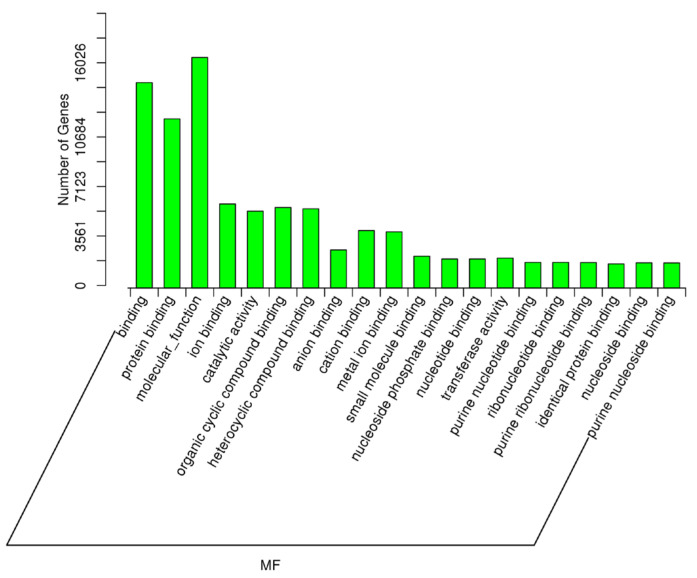
The GO enrichment analysis for the targets of differentially expressed miRNAs (RSPZNP vs. RSPZP) for molecular function (MF).

An additional key comparison in this study was made between individuals with rare spermatozoa who achieved pregnancy (RSPZP) and those who did not (RSPZNP). This analysis aimed to uncover the biological processes, cellular components, and molecular functions affected by differentially expressed miRNAs, providing insights into the molecular mechanisms that may contribute to reproductive success or failure. The most significantly enriched GO biological processes included cellular metabolic process, developmental process, nitrogen compound metabolic process, anatomical structure development, cellular component organization, primary metabolic process, multicellular organism development, regulation of biological quality, and metabolic process. The most enriched GO cellular component terms were intracellular, intracellular part, organelle, cytoplasm, membrane-enclosed lumen, nucleus, nucleoplasm, and non-membrane-bounded organelle. These results indicate that miRNA-regulated genes are primarily involved in intracellular structures, particularly within nuclear and cytoplasmic compartments, where they may influence gene expression. Finally, the most significantly enriched GO molecular function terms included protein binding, molecular function regulation, ion binding, catalytic activity, nucleoside phosphate binding, purine nucleotide binding, and transferase activity. These functions highlight the importance of protein interactions, enzymatic activity, and nucleotide metabolism (Figure 11, Figure 12 and Figure 13).

As in the previous analysis, we performed a pathway enrichment analysis to identify the biological pathways affected by the upregulated and downregulated miRNAs in the RSPZNP vs. RSPZP comparison. The enriched pathways associated with the gene targets of upregulated and downregulated miRNAs in RSPZNP are presented in Table 7.

A KEGG pathway analysis also identified the major biological pathways in which the target genes of differentially expressed miRNAs are involved among all comparisons. The most significantly enriched pathways, ranked by corrected *p*-values, are presented in Table 8. These enriched pathways highlight the key molecular mechanisms that regulate cellular metabolism, survival, and signal transduction.

## 3. Discussion

The role of miRNAs in spermatogenesis and male infertility has been widely investigated, revealing their critical involvement in gene regulation during germ cell development [27,29,35]. However, their specific expression patterns in azoospermic patients, particularly concerning testicular sperm presence and pregnancy outcomes, remain less explored. In this study, we characterized the small RNA expression profiles in testicular tissue samples from idiopathic azoospermic and cryptozoospermic patients who underwent testicular sperm extraction (TESE). Using small RNA sequencing, we identified differentially expressed (DE) miRNAs among the groups with high, rare, or no spermatozoa presence post-TESE, as well as between individuals with different pregnancy outcomes following assisted reproduction. To gain functional insights, we also conducted gene ontology (GO) and Kyoto Encyclopedia of Genes and Genomes (KEGG) enrichment analyses, which revealed key biological processes and molecular pathways associated with male infertility.

Our findings revealed extensive miRNA dysregulation across all samples, reinforcing the notion that changes in miRNA expression are intricately linked to azoospermia. Consistent with previous studies [28,36,37], we confirm that aberrant miRNA expression may contribute to spermatogenic failure, leading to male infertility, specifically azoospermia. However, we also observed distinct differences in miRNA expression between the sample groups, suggesting potential biological and clinical implications.

A key finding of this study was the consistent reduction of miRNA levels in the testicular samples with a high presence of spermatozoa compared to those with low or absent spermatozoa. This trend aligns with the hypothesis that miRNA-mediated gene regulation decreases as spermatogenesis progresses, possibly reflecting an optimized transcriptional landscape that supports mature sperm development. In contrast, the elevated miRNA expression in azoospermic samples with no spermatozoa may indicate incomplete spermatogenesis or represent a compensatory regulatory response to mitigate the disruptions in gene expression associated with spermatogenic failure. Several studies have reported that mature spermatozoa carry fewer miRNAs than earlier germ cells, further supporting the notion that miRNA activity decreases as germ cells differentiate into functional sperm [38,39]. Furthermore, our findings are consistent with previous studies indicating that miRNAs can serve as predictive biomarkers for the presence of sperm in testicular tissue [40,41,42,43], further highlighting their potential role in non-invasive diagnostic applications.

Regarding pregnancy outcomes, we identified differentially expressed miRNAs between the samples that resulted in pregnancy and those that did not, highlighting potential regulatory differences that may contribute to fertilization success. Notably, we observed a nearly equal distribution of upregulated and downregulated miRNAs. Although this suggests a complex regulatory landscape, studies that directly compare miRNA profiles in azoospermic patients who achieve a successful pregnancy vs. those who do not remain limited. Our findings underscore the potential of miRNA profiling as a tool for assessing testicular function and identifying novel biomarkers to predict sperm retrieval success and fertility potential in azoospermic patients.

In the following sections, we will discuss the specific miRNAs and pathways affected in these comparisons, providing further insights into their potential roles in spermatogenesis, sperm retrieval success, and reproductive outcomes.

### 3.1. miRNA-Mediated Dysregulation in NOSPZ vs. HIGHSPZ

The comparison between NOSPZ (no spermatozoa post-TESE) and HIGHSPZ (high sperm presence post-TESE) provides critical insights into the molecular differences that underpin successful versus impaired spermatogenesis. The stark contrast in sperm presence facilitates the examination of the key regulatory mechanisms that may differentiate functional spermatogenesis from spermatogenic failure.

The functional enrichment analysis of gene targets of differentially expressed miRNAs in the NOSPZ vs. HIGHSPZ comparison highlights key pathways involved in spermatogenesis, germ cell maintenance, and testicular function. The identified pathways suggest distinct regulatory disruptions between the two groups. Specifically, miRNAs upregulated in NOSPZ predominantly target the pathways related to cell survival, differentiation, and stress response, which may reflect an attempt to compensate for impaired spermatogenesis. In contrast, downregulated miRNAs are enriched in the pathways related to transcriptional regulation and signaling, potentially leading to dysregulated gene expression networks that are crucial for sperm production.

Specifically, regarding the pathways affected by upregulated miRNAs in NOSPZ, phosphoinositide-3-kinase/Akt (PI3K/AKT) signaling plays a pivotal role in germ cell survival, self-renewal of spermatogonial stem cells (SSCs), and spermatogenesis regulation [44,45]. Dysregulation of this pathway has previously been linked to defective sperm production, as AKT signaling supports SSC maintenance and proliferation and inhibits apoptosis in differentiating germ cells [29,46]. The observed upregulation of miRNAs targeting this pathway in NOSPZ suggests a potential repression of PI3K/AKT signaling, which could further contribute to germ cell depletion and impaired sperm production. Among the upregulated miRNAs found in NOSPZ, miR-26a and miR-26b have been implicated in the negative regulation of PI3K/AKT signaling [47,48], leading to reduced cell survival and increased apoptosis in germ cells. In porcine Sertoli cells, miR-26a has also been found to inhibit proliferation and promote apoptosis [49]. Additionally, the miR-125 family, including miR-125a and miR-125b, has been shown to modulate the PI3K/AKT/mTOR axis [50,51]. The overexpression of miR-125a and miR-125b in NOSPZ samples may inhibit AKT activation, leading to increased germ cell apoptosis and disrupted spermatogenesis. Similarly, estrogen receptors (ESRs), primarily ESR1 and ESR2, play a crucial role in testicular function and spermatogenesis, despite estrogens being traditionally associated with female reproductive biology [52]. ESR-mediated signaling is integral to the hormonal regulation of spermatogenesis, influencing germ cell proliferation, survival, and differentiation, as well as the maintenance of the blood–testis barrier and testis development [53,54]. Dysregulation of ESR signaling has previously been associated with altered testicular homeostasis [54,55], which may further compromise spermatogenic efficiency in azoospermic patients. Among the key regulators, members of the let-7 family, found to be upregulated in NOSPZ samples (let-7a-5p, let-7f-5p, let-7g-5p, let-7e-5p, let-7d-3p, let-7b-3p, and let-7c-3p), have been implicated in the regulation of estrogen receptor alpha (ERα) signaling [56]. Specifically, let-7b overexpression has been associated with reduced ERα expression, which in turn, can disrupt the transcriptional control of the genes that are essential for germ cell function [57]. Furthermore, elevated let-7b levels have been observed in oligospermic patients, reinforcing its potential association with male infertility [57,58]. Another significantly enriched pathway is FOXO-mediated transcription, which is known to regulate oxidative stress response, apoptosis, and cell cycle progression [59], all of which are essential for germ cell maintenance and spermatogenesis. In particular, the role of FOXO transcription factors in mouse spermatogenesis has been investigated, and *FOXO1* has been found to play a pivotal role in regulating various stages of spermatogenesis [60]. In the NOSPZ group, upregulated miRNAs appear to target components of this pathway, potentially leading to a dysregulated FOXO response, which could contribute to germ cell depletion and impaired spermatogenesis. For example, miR-27a-3p has been shown to suppress the FOXO pathway, thus reducing its ability to promote cell cycle arrest and apoptosis [61], processes that are crucial for germ cell homeostasis. Finally, the transforming growth factor-beta (TGFB) family plays a crucial role in testicular function by regulating germ cell development, Sertoli cell function, and overall spermatogenesis [62]. Disruptions in TGFB family signaling have been associated with male infertility, particularly in conditions characterized by spermatogenic failure [63]. Studies have shown that altered TGFB signaling can contribute to defects in germ cell maturation and the inability of SSCs to maintain an adequate pool for sustained spermatogenesis [63,64]. Among the miRNAs found to be upregulated in NOSPZ, several are regulators of TGFB signaling.

In contrast, downregulated miRNAs in NOSPZ are enriched in the pathways associated with transcriptional regulation and receptor-mediated signaling. Growth factor signaling plays a pivotal role in testicular development, germ cell survival, and the regulation of spermatogenesis [65]. Growth factor receptors, including EGFR, FGFR, and IGF1R, regulate essential testicular processes, sperm development and maturation, and their dysregulation has been linked to male infertility and testicular dysfunction [66,67,68,69,70]. The receptor tyrosine kinases (RTK) family, which was found to be an enriched pathway affected by downregulated miRNAs in NOSPZ, is also a major class of growth factor receptors. RTKs also play a crucial role in spermatogenesis by regulating germ cell proliferation, differentiation, and survival [71]. They are also important for maintaining communication between Sertoli cells and germ cells, ensuring the proper progression of spermatogenesis [72,73,74]. The observed downregulation of RTK signaling in NOSPZ samples suggests impaired RTK-mediated communication, which may contribute to defective sperm production and testicular dysfunction. In particular, Sertoli cells lacking RTKs show altered phagocytic activity toward apoptotic spermatogenic cells [73]. Among the downregulated miRNAs found in NOSPZ, miR-34c-5p has been associated with controlling c-Kit signaling [75]. Dysregulation of c-Kit signaling has been linked to impaired spermatogenesis and increased apoptosis [76]. Furthermore, miR-96-5p, which was also downregulated in NOSPZ, is significantly associated with receptor tyrosine kinase (RTK) signaling pathways [77,78]. Furthermore, second messengers, such as cyclic AMP (cAMP), calcium ions (Ca^2+^), inositol phosphates, and diacylglycerol (DAG), play critical roles in intracellular signaling cascades that regulate spermatogenesis, Sertoli cell function, and germ cell survival [79]. Downregulation of second-messenger signaling pathways in NOSPZ suggests a disrupted intracellular signaling environment that may contribute to defective sperm production. Additionally, in the NOSPZ vs. HIGHSPZ comparison, several pathways related to transcriptional control, including the generic transcription pathway, RNA polymerase II transcription, and gene expression regulation, were significantly downregulated. This trend suggests that, in NOSPZ, the increased miRNA expression leads to a greater suppression of transcription-related pathways, potentially impairing the gene expression programs necessary for efficient spermatogenesis.

**Figure 14:**
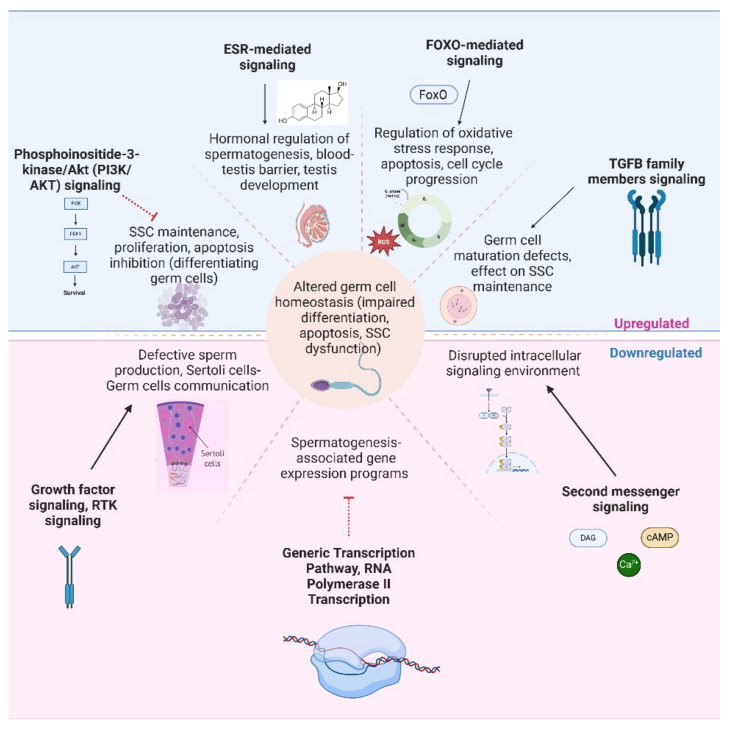
Upregulated and downregulated pathways in NOSPZ, along with mechanisms that lead to altered germ cell homeostasis. Created with biorender.com.

Overall, these findings suggest a coordinated disruption of key regulatory networks in NOSPZ, where upregulated miRNAs suppress essential pathways for cell survival and differentiation, with broader implications for germ cell homeostasis, while downregulated miRNAs compromise transcriptional activity and signal transduction, further impairing spermatogenic progression. This interplay between miRNA regulation, cell signaling, and gene expression control provides valuable insights into the molecular mechanisms contributing to the absence of spermatozoa in NOSPZ patients. A schematic representation is provided in Figure 14.

These findings collectively underscore the complexity of miRNA-mediated regulation in human spermatogenesis and suggest that altered expression patterns in NOSPZ are not merely reflective of impaired germ cell development but may actively contribute to the pathological processes leading to complete sperm absence. The convergence of upregulated miRNAs on key survival and differentiation pathways, such as PI3K/AKT, FOXO, TGFB, and estrogen receptor signaling, points to a multifaceted suppression of germ cell renewal, maturation, and endocrine regulation. At the same time, the downregulation of miRNAs targeting growth factor signaling, RTK activity, and transcriptional control further highlights the breakdown of crucial regulatory circuits in the testes. From a clinical perspective, these dysregulated miRNA signatures offer potential as non-invasive biomarkers for predicting TESE outcomes in azoospermic patients. Moreover, the identified pathways may provide therapeutic targets for restoring spermatogenesis or preventing further germ cell loss. The integration of these molecular insights with clinical phenotypes reinforces the role of miRNAs as both indicators and mediators of spermatogenic failure.

### 3.2. miRNA-Mediated Dysregulation in RSPZNP vs. RSPZP

The comparison between RSPZNP (rare spermatozoa presence—no pregnancy) and RSPZP (rare spermatozoa presence—pregnancy) provides valuable insights into the molecular differences that may affect fertilization success following TESE. A functional enrichment analysis of differentially expressed miRNAs revealed the key pathways related to immune signaling, growth factor regulation, and transcriptional control, indicating a dysregulated molecular environment in RSPZNP that may contribute to its unfavorable reproductive outcomes.

In RSPZNP, several upregulated miRNAs target pathways are crucial for immune balance, cell survival, and differentiation, potentially compromising the functional competence of retrieved spermatozoa. More specifically, the Interleukin-4 (IL-4) and Interleukin-13 (IL-13) signaling pathways are critical for maintaining immune homeostasis and mediating anti-inflammatory responses within the male reproductive tract [80]. IL-4 plays a pivotal role in modulating local immune responses, and its dysregulation has been linked to compromised sperm function and quality [81]. Similarly, IL-13 is implicated in promoting inflammatory conditions that can impair sperm transport and overall function [82]. Our data indicate that differentially expressed miRNAs negatively regulate these pathways in the testis, suggesting an altered immunological microenvironment that may adversely affect reproductive function. In particular, miR-155-5p, an miRNA known for its pro-inflammatory effects [83], was prominently upregulated. In the testicular context, elevated levels of miR-155-5p could further suppress key components of the IL-4/IL-13 pathways, thereby shifting the local balance toward a pro-inflammatory state. Previous studies have confirmed the association of this miRNA with inflammation-related male infertility [84]. Therefore, this immune shift may undermine the immune-privileged status of the testis, disrupt spermatogenic processes, and ultimately compromise fertility. Given the essential role of cytokine signaling in protecting germ cells from excessive immune activation [80], its suppression could contribute to suboptimal sperm maturation and increased inflammatory responses, highlighting a potential immune-mediated mechanism underlying impaired fertility and poor reproductive outcomes. Intracellular signaling mediated by a second messenger, including cyclic AMP, calcium ions, and inositol phosphates, is fundamental for coordinating the complex cellular processes underlying testicular function, spermatogenesis, and the acrosome reaction [79,85]. Our small RNA sequencing analysis revealed a broad spectrum of differentially expressed miRNAs predicted to target key components of these signaling cascades. Among these, miR-324-5p targets genes involved in several pathways, while miR-132-5p has been found to influence apoptosis and calcium signaling pathways in other cell types [86] Calcium ions are crucial for several fertilization-associated events, including sperm capacitation and motility, the acrosome reaction, and the transient calcium influx that triggers egg activation [87,88]. In this context, miR-132-5p might not only affect the testicular microenvironment but could also have downstream effects on sperm functionality and fertilization capacity. Furthermore, as described in the previous section, the upregulation of miRNAs targeting the PIP3/AKT signaling pathway can lead to reduced cell survival and increased apoptosis in germ cells, potentially resulting in defects in sperm quality and function. Among the DE miRNAs, hsa-miR-199b-5p, hsa-miR-424-5p, and hsa-miR-486-5p target genes of the above pathway. Additionally, altered TGFB signaling, due to increased miRNA-mediated repression, can also contribute to defects in germ cell maturation, further supporting the notion of an impaired spermatogenic niche in RSPZNP. Finally, the increased miRNA-mediated suppression of receptor tyrosine kinase (RTK) signaling, a pathway involved in cell proliferation and survival, suggests a further impairment of testicular cell function and maintenance. Hsa-miR-128-3p and hsa-miR-7-5p are two of the upregulated miRNAs in RSPZNP that target genes of the RTK signaling pathway.

In contrast, the downregulated miRNAs in RSPZNP indicate a significant loss of regulatory control over hormonal signaling and transcriptional regulation in these samples. More specifically, ESR-mediated signaling, which plays an essential role in germ cell maintenance and hormone-dependent regulation of spermatogenesis, as mentioned previously, was significantly disrupted. Reduced ESR activity has been associated with decreased sperm motility, viability, and capacitation, underscoring its importance beyond sperm production and into functional competence [89,90,91]. The observed downregulation of ESR-mediated signaling in RSPZNP, a group characterized by failed pregnancy outcomes, suggests that estrogenic regulation may be a crucial determinant of sperm functionality rather than merely its presence. Among the DE miRNAs identified to target genes of this pathway are hsa-let-7b-5p, hsa-miR-146a-5p, hsa-miR-146b-5p, hsa-miR-22, and hsa-miR-206. In addition, signaling by nuclear receptors was affected by DE miRNAs. Nuclear receptor signaling plays a fundamental role in male reproductive function by modulating the hormonal responses that are essential for spermatogenesis [92,93]. Among the nuclear receptors, the androgen receptor (AR) is critical for Sertoli cell function, ensuring proper support for germ cell maturation and the maintenance of the blood–testis barrier [94,95]. Similarly, retinoic acid receptors (RARs) are essential for spermatogonial differentiation, and deficiencies in this pathway lead to impaired sperm production and epididymal dysfunction [96,97]. Other nuclear receptors associated with impaired spermatogenesis and hormonal regulation in males include PPAR, VDR, and FXRα [93]. Thus, alterations in nuclear receptor signaling can interfere with hormonal homeostasis and contribute to abnormal spermatogenesis. In the RSPZNP group, the downregulation of nuclear receptor signaling suggests a potential impairment in hormonal regulation. This disruption could contribute to defective capacitation, motility, or fertilization capacity, ultimately affecting reproductive success. Finally, the downregulated miRNAs target transcriptional pathways, including RNA polymerase II transcription and the generic transcription pathway. The observed downregulation of these pathways in RSPZNP suggests a broader impairment in transcriptional activity, which may compromise the expression of the genes necessary for normal sperm function and fertilization potential. Furthermore, this loss of transcriptional regulation may indicate an inability to compensate for spermatogenic defects, exacerbating functional deficiencies in the spermatozoa retrieved from RSPZNP patients.

Taken together, these findings suggest that pregnancy failure in RSPZNP patients may be attributed to impaired transcriptional regulation, disrupted hormone-responsive signaling, and altered immune and survival pathways, all of which compromise sperm function and fertilization potential.

These observations highlight that, even in cases where rare spermatozoa can be retrieved (RSPZNP), specific miRNA expression patterns may compromise their fertilization capacity by targeting the pathways essential for immune balance, hormonal regulation, and transcriptional control. The dysregulation of ESR and nuclear receptor signaling suggests that subtle hormonal imbalances may affect not only sperm production but also post-testicular sperm functionality, such as capacitation and acrosome reaction, which are crucial for successful fertilization. Furthermore, the suppression of cytokine and second-messenger signaling points to a disturbed testicular microenvironment, potentially contributing to sperm dysfunction at both the structural and molecular level. From a translational standpoint, these findings suggest that miRNA profiling may help identify patients whose sperm, although present, are functionally compromised and less likely to achieve fertilization or pregnancy. Ultimately, the integration of miRNA-based biomarkers with clinical outcomes may pave the way for more personalized prognostic tools in the management of male infertility and assisted reproduction strategies.

### 3.3. miRNAs as Male Infertility Biomarkers and the Role of miR-34c-5p

Conventional semen analysis, while essential for assessing male fertility, often fails to fully explain cases of infertility or accurately predict the success of assisted reproductive technologies (ART) [98]. Although morpho-functional sperm characteristics provide valuable clinical information, they do not capture the molecular disruptions that may impair spermatogenesis, fertilization potential, or embryo development. As a result, there is a critical need for more sensitive and reliable biomarkers that can enhance male fertility evaluation and improve ART outcomes.

Our study aimed to address this gap by investigating small RNAs as potential biomarkers for predicting spermatozoa presence in testicular samples following TESE. Identifying molecular signatures associated with spermatogenesis impairment could aid in patient stratification for TESE success, ultimately leading to more personalized fertility treatments. However, research on small RNAs in this context remains limited, particularly regarding their relationship to pregnancy outcomes and embryo quality.

In this regard, the study by Conflitti et al. (2023) [99] provides valuable insight into the association between sperm DNA fragmentation (SDF), sperm-borne miR-34c-5p and miR-449b-5p levels, and ART success parameters. Their findings highlight that higher levels of miR-34c-5p relative to miR-449b-5p were associated with a 14-fold increase in the probability of obtaining viable embryos, reinforcing its role in sperm competence and early embryogenesis. Our findings align with these observations, as we identified a significant downregulation of miR-34c-5p in NOSPZ samples compared with HIGHSPZ samples, suggesting its involvement in spermatogenesis failure. Additionally, miR-34c-5p has been widely reported to be downregulated in men with impaired spermatogenesis, including those with oligozoospermia, asthenozoospermia, teratozoospermia, oligoasthenoteratozoospermia, and idiopathic male infertility, and also in the seminal plasma of obstructive and nonobstructive azoospermia [100,101,102,103]. Similarly, Fang et al. (2019) [104] investigated and compared the microRNA profiles of NOA patients with successful and unsuccessful sperm retrieval. They found that the two groups had 180 differentially expressed microRNAs, with the miR-34 cluster being among the most downregulated in testicular biopsies and the seminal plasma of the unsuccessful sperm retrieval group [104]. Interestingly, our study also revealed that miR-34c-5p was upregulated in the RSPZP samples compared to the RSPZNP samples, further supporting its potential role in successful fertilization and embryo viability. This finding is consistent with a study by Cui et al. (2015) [105] that showed that sperm-borne miR-34c was associated with better embryo quality at day 3 and higher implantation, pregnancy, and birth rates.

The biological mechanisms regulating miR-34c-5p remain an active area of investigation. Previous studies have shown that, in the male reproductive system, the microRNA-34/449 family miRNA inhibits proliferation and promotes germ cell survival, differentiation, and apoptosis of excess and defective germ cells [106]. Members of the miR-34/449 family are crucial for normal testicular functionality and successful spermatogenesis [99]. According to a review by Pantos et al. (2021) [106], the dysregulation of these miRNAs can lead to impaired ciliogenesis in the efferent ductules epithelium, resulting in sperm aggregation, defective reabsorption of seminiferous tubular fluids, and ultimately, spermatogenic failure and male infertility. Additionally, these miRNAs exhibit significant antioxidant and anti-apoptotic properties, contributing to testicular homeostasis [106]. 

Taken together, these findings emphasize the urgent need for novel diagnostic and predictive tools beyond conventional semen analysis. Given its strong association with spermatogenesis, embryo viability, and pregnancy outcomes, miR-34c-5p should be further explored as a potential biomarker for male fertility assessment and ART success prediction. Furthermore, the potential association between miR-449a/b/c and miR-34b/c suggests a possible synergistic action, the molecular mechanisms of which are unknown but would be interesting to study in the future. Thus, by integrating molecular profiling approaches with routine fertility assessments, future studies could refine male infertility diagnostics, enhance ART success rates, and improve clinical management strategies for infertile patients.

### 3.4. Potential Functional Roles of Novel miRNAs Identified

Our study identified several novel miRNAs that were differentially expressed across key infertility subgroups, highlighting their potential involvement in spermatogenesis and sperm function.

Notably, the comparison between RSPZNP vs. RSPZP revealed a substantial number of novel miRNAs, indicating that previously uncharacterized regulatory molecules may not only contribute to sperm presence but, more importantly, influence sperm functionality, potentially impairing fertilization. To further explore the biological relevance of these novel miRNAs, we performed target prediction followed by integrative analysis, evaluating the potential miRNA–target gene interactions. Similar to differentially expressed known miRNAs, our analysis identified numerous genes strongly implicated in cell survival and germ cell apoptosis, particularly through the PIP3/AKT signaling pathway, as well as other critical pathways such as TGFB signaling and receptor tyrosine kinase (RTK) signaling. More importantly, several novel miRNAs appear to target the genes associated with a proinflammatory state, suggesting that both novel and known miRNAs may contribute to an altered testicular microenvironment, ultimately leading to impaired testicular function and disrupted spermatogenesis. Additionally, some novel miRNAs were detected exclusively in the RSPZNP samples and were entirely absent in the RSPZP, indicating that these candidate miRNAs may exhibit high specificity, potentially making them promising diagnostic biomarkers for rare sperm presence without fertilization potential.

Furthermore, in the HIGHSPZ vs. CRYPTO comparison, novel_30 exhibited the most pronounced differential expression, with a dramatic log_2_ fold change of −94.856 (q-value = 1.05 × 10^−57^), indicating its significant downregulation in CRYPTO. Given that cryptozoospermia is characterized by an extremely low number of spermatozoa in the ejaculate, often associated with impaired spermatogenesis, the marked suppression of novel_30 suggests a potential regulatory role in germ cell development and sperm production. To further investigate its functional relevance, we performed target prediction analysis, which revealed that novel_30 is predicted to regulate several key genes implicated in apoptosis, spermatogenesis, and hormonal signaling. Among its predicted targets, *BCL2L11* (*BIM*) is a well-known pro-apoptotic gene involved in germ cell survival [107], suggesting that novel_30 may modulate the apoptosis-related pathways that are essential for spermatogenesis. Additionally, novel_30 is predicted to target *KIT*, a receptor tyrosine kinase crucial for spermatogonial stem cell maintenance and differentiation [108], implying a potential role in regulating early spermatogenic processes. The significant downregulation of novel_30 in CRYPTO, along with its predicted regulatory impact on these pathways, indicates that its dysregulation may contribute to impaired sperm production and testicular dysfunction.

While bioinformatics predictions provide valuable insights, experimental validation is necessary to determine the precise roles of these novel miRNAs. Future studies should focus on confirming miRNA–mRNA interactions to establish direct regulatory relationships and delineate their functional impact. Functional assays, including luciferase reporter assays, in vitro models, and in vivo validation, will be critical to verifying their biological role in male infertility. Furthermore, expanding these findings to larger patient cohorts could help determine the diagnostic and prognostic utility of novel miRNAs, refining their potential as biomarkers for male infertility assessment and treatment stratification.

### 3.5. Strengths and Limitations—Future Directions

The present study has several notable strengths and limitations. A key strength is its focus on multiple clinically relevant comparisons, including the presence versus absence of sperm and pregnancy versus non-pregnancy outcomes. This comprehensive approach facilitates the identification of candidate miRNAs that may serve as biomarkers for predicting sperm retrieval success and fertility potential in azoospermic patients. Moreover, by incorporating a diverse range of phenotypes, our study offers valuable insights into the distinct molecular mechanisms underlying sperm deficiencies while also distinguishing shared and unique miRNA signatures. Additionally, the use of well-defined patient groups minimizes variability, thereby enhancing the robustness and clinical relevance of our findings.

However, certain limitations should be acknowledged. First, while the sample size was sufficient to detect differential miRNA expression, it may limit the generalizability of our findings to a broader population. The challenge of obtaining testicular biopsy samples contributed to this limitation, emphasizing the need for larger cohorts and independent validation to confirm the clinical utility of the identified miRNA signatures as potential biomarkers. More specifically, a small sample size can affect statistical power, increasing the risk of both false positives and false negatives in a differential expression analysis. Although our findings align with previous studies, replication in larger, well-powered cohorts is essential to ensure the robustness of our results. Additionally, variability in miRNA expression across different subgroups of infertile patients may not be fully captured in a limited dataset, further underscoring the importance of expanding the sample size in future studies. To strengthen the clinical applicability of our findings, multi-center studies with larger and more diverse patient populations should be conducted. Such studies would not only enhance the statistical power but also allow for subgroup analyses to assess whether specific miRNA expression patterns are associated with distinct infertility phenotypes. Second, although pathway enrichment analyses provide valuable functional insights, experimental validation is essential to establish direct causal relationships between specific miRNAs and their predicted targets in the context of spermatogenesis. Another main limitation of this study is the use of pooled RNA samples for small RNA sequencing. Pooling was selected due to logistical and financial constraints and to include a larger number of participants within the study’s scope. However, while pooling is a common strategy in exploratory studies to optimize resources, it may reduce biological and technical variability compared to sequencing individual samples. This limitation affects the ability to capture interindividual differences and may influence the generalizability of our findings. Thus, future studies should prioritize sequencing at the individual level to enhance resolution and better detect subtle differences in miRNA expression. A more refined approach that incorporates single-sample sequencing would provide greater insight into patient-specific miRNA profiles and improve the identification of potential biomarkers. Additionally, while our study identified aberrantly expressed miRNAs, we acknowledge the absence of qRT-PCR validation as another limitation. Due to sample constraints, qRT-PCR validation could not be performed in the current study. However, independent validation remains a crucial step in confirming differentially expressed miRNAs and ensuring the reproducibility of our findings. Future research should incorporate targeted qRT-PCR assays to validate the candidate miRNAs reported to have differential expression in the present study and further assess their clinical relevance in male infertility. Additionally, future studies integrating multi-omics approaches, such as proteomics and metabolomics, could offer a more comprehensive understanding of the molecular mechanisms underlying spermatogenic failure and pregnancy outcomes in azoospermic patients.

Furthermore, one key aspect that remains unexplored is the potential molecular distinctions between HIGHSPZ and RSPZP. Our correlation analysis indicated that these groups exhibit the lowest similarity, suggesting possible biological differences. However, due to the lack of fertility outcome data for HIGHSPZ, we deliberately refrained from performing a differential expression analysis between them, as such a comparison would be difficult to interpret and could lead to inconclusive results. Future studies integrating clinical follow-up data, particularly regarding reproductive outcomes in individuals classified as HIGHSPZ, could offer a more comprehensive understanding of whether the molecular differences between HIGHSPZ and RSPZP are associated with fertility potential. Longitudinal studies tracking these patients could provide the necessary context to determine whether specific transcriptomic variations correlate with successful conception. Similarly, a future study including patients with a high spermatozoa presence and distinguishing between those who achieved pregnancy and those who did not could further contribute to biomarker development and a better understanding of the mechanisms of male infertility.

Furthermore, while our study provides novel insights into the small RNA profiles in testicular tissues of infertile men and their potential as biomarkers for predicting sperm retrieval success after TESE, future research should aim to broaden the scope of these findings by exploring the implications of this biomarker approach across a wider range of conditions requiring TESE. A notable avenue for future research involves men with bladder neck sclerosis (BNS), an anatomical anomaly in which scar tissue or narrowing at the bladder neck obstructs normal semen outflow. In many cases, this dysfunction leads to low-volume or absent antegrade ejaculation and can result in obstructive azoospermia, i.e., a total lack of sperm in the ejaculate despite normal spermatogenesis [109]. Surgical intervention, such as transurethral incision of the bladder neck, often alleviates urinary flow issues but poses a significant risk of retrograde ejaculation or anejaculation [110]. Consequently, men with BNS who wish to preserve fertility may benefit from early sperm cryopreservation, and those who do not undergo preoperative banking often require TESE if they develop severe post-surgical ejaculatory dysfunction [110,111]. Importantly, small RNA signatures in testicular biopsies from these patients may differ from those observed in non-obstructive azoospermia (NOA), thus warranting further investigative studies.

Another relevant procedure for men with obstructive pathologies is transurethral resection of the ejaculatory ducts (TURED). This technique primarily addresses ejaculatory duct obstruction, but it can also benefit individuals who have scarring near the bladder neck that functionally blocks ejaculatory flow. Although TURED can restore antegrade emission in certain cases [112], some patients continue to experience azoospermia or produce insufficient sperm for natural conception. In these scenarios, TESE remains a viable option, with reported near-complete retrieval success in obstructive etiologies [109]. Similar to BNS, cryopreservation of surgically retrieved sperm is strongly advised to minimize the need for repeated invasive procedures [110].

Beyond bladder neck sclerosis and ejaculatory duct obstruction, there are other conditions in which TESE is necessary to circumvent anatomical barriers to sperm transport. These include a congenital bilateral absence of the vas deferens (CBAVD) and postsurgical cases, such as vasectomy. In all such examples of obstructive azoospermia, the prognosis for sperm retrieval is usually higher than for NOA [113]. Consequently, investigating small RNA profiles in a variety of obstructive scenarios could reveal important differences in molecular signatures that may refine the current predictive models of TESE success. Indeed, prior evidence suggests that molecular signatures, including small RNAs, may vary across azoospermic samples of varying etiologies, underscoring the need for multi-cohort analyses [36,37,114].

The present data indicate that small RNA dysregulation in azoospermic testes might predict sperm retrieval success, but further studies must consider other relevant clinical subgroups. By including BNS patients and those undergoing TURED or other reconstructive surgeries, researchers can test whether identified small RNA biomarkers remain robust in different obstructive contexts. Evaluating whether specific small RNAs correlate with the severity of bladder neck pathology, or with the extent of ejaculatory dysfunction, would also be a logical next step. Ultimately, these efforts will broaden the applicability of small RNA-based biomarkers and improve individualized treatment strategies for men with impaired fertility due to a range of anatomical and functional causes.

## 4. Materials and Methods

### 4.1. Patient Recruitment

The present study included forty patients diagnosed with azoospermia or cryptozoospermia who underwent testicular biopsy as part of their assisted reproduction treatment at the Embryolab Fertility Clinic (Thessaloniki, Greece). Written informed consent was obtained from all participants, and the study was approved by the Ethics Committee of the University of Thessaly.

Azoospermia was diagnosed after at least two semen analyses conducted according to World Health Organization (WHO) guidelines. A comprehensive diagnostic process was followed, including a medical history assessment, physical examination, and scrotal imaging. All participants also completed a detailed questionnaire about their medical history, lifestyle factors, etc. (File S1). Further investigations included genetic tests for karyotype, DF508 mutation, Y-chromosome microdeletions, and serum hormone measurements (follicle-stimulating hormone, luteinizing hormone, and testosterone). Only patients in whom no identifiable cause of male infertility was found were included in this study.

During testicular sperm extraction (TESE), experienced urologists recovered multiple tissue samples from both testicles, which were mechanically dissected and microscopically examined for the presence of mature spermatozoa. Biopsies were categorized based on the abundance of spermatozoa as follows: (i) “No presence”—no spermatozoa observed after at least 30 min of searching under an inverted microscope by three experienced operators; (ii) “Rare presence”—spermatozoa found after more than 10 min of searching; and (iii) “High presence”—spermatozoa identified within 2 min of searching [115]. Furthermore, in this study, cases of cryptozoospermia, in which TESE was also performed to achieve assisted reproduction, were also included. For small RNA sequencing, a small portion of each testicular tissue sample was collected in sterile Falcon tubes and stored at −80 °C until shipment on dry ice to the Laboratory of Genetics, Comparative and Evolutionary Biology (University of Thessaly, Larissa, Greece), for RNA extraction and further processing of the samples. The patients were followed-up to assess pregnancy status, and the samples were further categorized based on spermatozoa presence in the testes tissue and pregnancy outcomes following assisted reproduction techniques, as follows: Cryptozoospermia (*n* = 8), no presence of spermatozoa (*n* = 8), rare presence of spermatozoa—no pregnancy (*n* = 8), rare presence of spermatozoa—pregnancy (*n* = 8), and high presence of spermatozoa (*n* = 8).

### 4.2. RNA Extraction and Sample Preparation

Total RNA was extracted from 40 testicular tissue samples using the miRNeasy Mini Kit (Qiagen, USA) according to the manufacturer’s protocol. RNA quality was evaluated using agarose gel electrophoresis, and the RNA quantity was measured using a Qubit 2.0 fluorometer (Thermo Fisher Scientific, Waltham, MA, USA) and the Qubit microRNA Assay Kit (Thermo Fisher Scientific, Waltham, MA, USA). Only samples with high RNA quality and a concentration exceeding 80 ng/μL were included in this study.

Ten sequencing pools were then created to facilitate downstream analyses. To ensure reproducibility and robustness in small RNA profiling, we generated two biological replicates (pools) per group. The samples were not pooled randomly. Instead, pooling was conducted strategically to minimize variability and account for potential confounding factors. More specifically, we ensured that the two pools within each group had similar characteristics, such as age, health habits, and clinical parameters, to enhance comparability. This approach ensured that each pool was representative of its respective group while reducing the impact of individual variability on small RNA expression profiles. Specifically, two pools were generated from azoospermic samples with no spermatozoa (NOSPZ), two pools for samples with rare spermatozoa presence and no pregnancy (RSPZNP), two pools for samples with rare spermatozoa presence and successful pregnancy (RSPZP), two pools for samples with high spermatozoa presence (HIGHSPZ), and two pools for cryptozoospermia (CRYPTO). Each pool contained RNA from four individual samples. RNAs were mixed equimolarly for each pool.

### 4.3. Small RNA Library Construction and Sequencing

Following preparation, RNA samples were shipped to Novogene Co. (Cambridge, UK), where small RNA libraries were constructed. Briefly, 3′ and 5′ adapters were ligated to the respective ends of the small RNAs. First-strand cDNA synthesis was performed after hybridization with a reverse transcription primer, followed by PCR amplification to generate double-stranded cDNA libraries. The libraries were purified and size-selected to retain inserts between 18–40 bp, preparing them for sequencing on an Illumina HiSeq 2500 platform (Illumina, San Diego, CA, USA).

After sequencing, the raw data were subjected to rigorous quality control. Reads containing adapters, low-quality bases, or sequences shorter than 18 nucleotides were removed using Trimmomatic [116]. The resulting clean reads were mapped to the human reference genome (GRCh37/hg19) using Bowtie 2 [117,118]. MiRNA identification was performed using miRBase20.0 [119] as a reference, with modified mirdeep2 [120] and the sRNA toolbox [121] utilized to predict potential miRNAs and generate the secondary structures. Reads were further mapped to the RepeatMasker (http://www.repeatmasker.org, accessed on 16 February 2025) and Rfam [122] databases to annotate and filter out repeat sequences and other non-coding RNAs, including ribosomal RNA (rRNA), transfer RNA (tRNA), small nucleolar RNA (snoRNA), small nuclear RNA (snRNA), and small cytoplasmic RNA (scRNA). Novel miRNAs were predicted by integrating miREvo [123] and mirdeep2 [120], analyzing the secondary structure, Dicer cleavage sites, and the minimum free energy of unannotated small RNA tags. Finally, to ensure the unique annotation of the reads, the following annotation ranking was applied: known miRNA > rRNA > tRNA > snRNA > snoRNA > repeat > gene > NAT-siRNA > novel miRNA > ta-siRNA.

### 4.4. Analysis of Differentially Expressed miRNAs and Functional Enrichment of Their Target Genes

The expression levels of the miRNAs were first normalized using transcripts per million (TPM). The TPM value is calculated by the actual reads of each miRNA×10^6^ divided by the total reads of all miRNAs. Differential expression analysis was performed using the DESeq v2.0 R package [124] in all pairwise comparisons between the five conditions studied. The *p*-values were adjusted using the q-value method [125], and miRNAs with an adjusted *p*-value (q-value) < 0.01 and |log_2_ (foldchange)| > 1 were considered significantly differentially expressed.

Subsequently, to investigate the potential biological roles of differentially expressed miRNAs, target gene predictions were conducted using miRanda [126]. Gene ontology (GO) enrichment analysis [127,128] and pathway enrichment analysis using the Kyoto Encyclopedia of Genes and Genomes (KEGG) database [129,130] were performed on the predicted target genes. Pathway significance (*p*-value < 0.05) was evaluated using the hypergeometric test, with the *p*-values adjusted for multiple testing using the false discovery rate (FDR) correction. Statistical enrichment of the target gene candidates in the KEGG pathways was also tested using the KOBAS software 2.0 [131].

## 5. Conclusions

This study provides a preliminary overview of the role of miRNA dysregulation in cryptozoospermic and azoospermic patients, highlighting the distinct molecular differences associated with sperm presence and pregnancy outcomes. While the findings offer valuable insights and suggest potential molecular markers of male infertility, they should be interpreted with caution due to several limitations. Notably, the absence of qRT-PCR validation represents a significant constraint in confirming the expression levels of the identified miRNAs. Future studies should prioritize experimental validation using targeted qRT-PCR assays to strengthen the reliability of the results and to further explore the clinical relevance of these miRNAs in the context of male infertility. Despite these limitations, our findings lay the groundwork for future investigations that aim to clarify the molecular underpinnings of male infertility and identify robust diagnostic biomarkers.

## Figures and Tables

**Table 1:** Characteristics of participants included in the present study. *p*-values (*p* > 0.05) indicate no statistically significant differences between the studied groups.

	Cryptozoospermia —CRYPTO (*n* = 8)	No Presence of Spermatozoa—NOSPZ (*n* = 8)	Rare Presence of Spermatozoa—No Pregnancy—RSPZNP (*n* = 8)	Rare Presence of Spermatozoa—Pregnancy—RSPZP (*n* = 8)	High Presence of Spermatozoa—HIGHSPZ (*n* = 8)	*p*-Value
**Age**	31–44 Mean = 37.5 (SD = 6.45)	29–39 Mean = 33.75 (SD = 4.57)	24–45 Mean = 34.5 (SD = 9.04)	31–41 Mean = 36.25 (SD = 4.27)	n28–41 Mean = 34.5 (SD = 6.45)	0.917 (ANOVA)
**Body Mass Index (BMI)**	22.8–28.1 Mean = 25.9 (SD = 2.64)	22.9–33.2 Mean = 29.38 (SD =4.56)	20.6–34 Mean = 27.22 (SD = 5.48)	18.5–30.1 Mean = 25.55 (SD = 5.27)	22.5–30.8 Mean = 27.88 (SD = 4.51)	0.769 (ANOVA)
**Smoking**	50% No, 50% Yes	50% No, 50% Yes	60% No, 40% Yes	50% No, 50% Yes	60% No,40% Yes	0.870 (chi-square test)
**Alcohol Consumption**	100% ≤ 2 drinks/week	80% ≤ 2 drinks/week	80% ≤ 2 drinks/week	100% ≤ 2 drinks/week	80% ≤ 2 drinks/week	0.671 (chi-square test)

**Table 2:** Summary of sequencing data of all samples.

Sample Name	Total Reads	Clean Reads	Percentage	Mapping Rate
NOSPZ1	22,823,882	21,824,598	95.6%	69.6%
NOSPZ2	23,245,844	21,639,163	93.1%	71.9%
RSPZNP1	23,522,135	22,201,524	94.4%	73.43%
RSPZNP2	23,968,227	21,964,941	91.6%	74.1%
RSPZP1	23,808,843	22,429,360	94.2%	79.34
RSPZP2	23,072,834	21,966,578	95.2%	77.8%
HIGHSPZ1	21,980,184	20,449,851	93%	78.00%
HIGHSPZ2	21,604,874	20,557,389	95.2%	76.5%
CRYPTO1	22,930,151	21,852,645	95.3%	75.3%
CRYPTO2	21,886,205	20,179,995	92.2%	72.8%

**Table 3:** Top 10 differentially expressed (DE) miRNAs in the comparison of NOSPZ vs. HIGHSPZ. The table includes information on dysregulation, fold changes, and adjusted *p*-values (*q*-values).

miRNA	Dysregulation	Log_2_ Fold Change	*q*-Value
hsa-miR-450b-5p	Up	95.876	1.50 × 10^−52^
hsa-miR-615-3p	Up	91.265	1.78 × 10^−39^
hsa-miR-129-1-3p	Up	84.732	8.96 × 10^−26^
hsa-miR-135a-5p	Up	82.785	1.41 × 10^−22^
hsa-miR-506-5p	Up	82.785	1.41 × 10^−22^
hsa-miR-29b-3p	Up	80.721	1.49 × 10^−19^
hsa-miR-433-3p	Up	76.936	7.11 × 10^−15^
hsa-miR-708-3p	Up	76.936	7.11 × 10^−15^
hsa-miR-891a-5p	Up	76.693	1.31 × 10^−14^
hsa-miR-494-3p	Up	76.196	4.54 × 10^−14^

**Table 4:** Top 10 differentially expressed (DE) miRNAs in the comparison HIGHSPZ vs. CRYPTO. The table includes information on dysregulation, fold changes, and adjusted *p*-values (*q*-values).

miRNA	Dysregulation	Log_2_ Fold Change	*q*-Value
novel_30	Down	−94.856	1.05 × 10^−57^
hsa-miR-1-3p	Down	−92.529	2.15 × 10^−50^
hsa-miR-193b-5p	Down	−86.315	9.65 × 10^−35^
hsa-miR-125b-2-3p	Down	−82.529	1.76 × 10^−27^
hsa-miR-891a-5p	Down	−80.185	1.14 × 10^−23^
hsa-miR-3614-5p	Down	−78.379	4.17 × 10^−21^
hsa-miR-378c	Down	−78.379	4.17 × 10^−21^
hsa-miR-708-3p	Down	−78.379	4.17 × 10^−21^
hsa-miR-7706	Down	−78.379	4.17 × 10^−21^
hsa-miR-129-1-3p	Down	−77.384	8.79 × 10^−20^

**Table 5:** Top 10 differentially expressed (DE) miRNAs in the comparison RSPZNP vs. RSPZP. The table includes information on dysregulation, fold changes, and adjusted *p*-values (*q*-values).

miRNA	Dysregulation	Log_2_ Fold Change	*q*-Value
hsa-miR-146b-3p	Down	−76.007	6.32 × 10^−19^
hsa-miR-1247-5p	Down	−75.045	9.33 × 10^−18^
hsa-miR-659-5p	Down	−71.059	1.68 × 10^−13^
hsa-miR-206	Down	−69.684	2.88 × 10^−12^
hsa-miR-450b-5p	Up	69.255	8.73 × 10^−13^
hsa-miR-3148	Down	−65.534	4.93 × 10^−9^
hsa-miR-6511b-5p	Down	−64.539	2.23 × 10^−8^
novel_41	Down	−62.315	5.00 × 10^−7^
novel_45	Down	−62.315	5.00 × 10^−7^
hsa-miR-369-3p	Up	60.775	8.89 × 10^−7^

**Table 6:** Top five enriched pathways of gene targets of upregulated and downregulated miRNAs in NOSPZ (vs. HIGHSZP).

Upregulated	Downregulated
PI3K/AKT Signaling	Signaling by receptor tyrosine kinases
ESR-mediated signaling	Generic transcription pathway
PIP3-activated AKT signaling	RNA polymerase II transcription
FOXO-mediated transcription	Diseases of signal transduction by growth factor receptors and second messengers
Signaling by TGFB family members	Gene expression (transcription)

**Table 7:** Top five enriched pathways of gene targets of upregulated and downregulated miRNAs in RSPZNP (vs. RSPZP).

Upregulated	Downregulated
Interleukin-4 and Interleukin-13 signaling	ESR-mediated signaling
PIP3 activates AKT signaling	Signaling by nuclear receptors
Signaling by TGFB family members	Generic transcription pathway
Intracellular signaling by second messengers	RNA polymerase II transcription
Signaling by receptor tyrosine kinases	Gene expression (transcription)

**Table 8:** KEGG enrichment analysis for the target genes of differentially expressed miRNAs across all comparisons.

KEGG Term	Corrected *p*-Value
Metabolic pathways	1.20602675899 × 10^−72^
PI3K-Akt signaling pathway	1.87222793907 × 10^−20^
Cytokine–cytokine receptor interaction	2.21189356028 × 10^−15^
MAPK signaling pathway	8.85018316676 × 10^−15^

## Data Availability

The dataset is available upon request from the authors.

## Supplementary Materials

The following supporting information can be downloaded at https://www.mdpi.com/article/10.3390/ijms26083537/s1: File S1: Patient questionnaire used for data collection in this study. The questionnaire includes demographic, lifestyle, medical history, and reproductive health-related questions to assess potential factors influencing male fertility.

## Abbreviations

The following abbreviations are used in this manuscript:

AR	Androgen Receptor
ART	Assisted Reproductive Technologies
BMI	Body Mass Index
CRYPTO	Cryptozoospermia
DAG	Diacylglycerol
DE	Differentially Expressed
ESR	Estrogen Receptor
GO	Gene Ontology
HIGHSPZ	High presence of Spermatozoa
ICSI	Intra-Cytoplasmic Sperm Injection
IL-4	Interleukin-4
IL-13	Interleukin-13
IVF	In Vitro Fertilization
KEGG	Kyoto Encyclopedia of Genes and Genomes
miRNA	MicroRNA
NOA	Non-obstructive Azoospermia
NOSPZ	No Presence of Spermatozoa
OA	Obstructive Azoospermia
PI3K	Phosphoinositide-3-Kinase
RAR	Retinoic Acid Receptor
RSPZNP	Rare Presence of Spermatozoa—No Pregnancy
RSPZP	Rare Presence of Spermatozoa—Pregnancy
RTK	Receptor Tyrosine Kinases
SSC	Spermatogonial Stem Cell
sRNA	Small RNA
TESA	Testicular Sperm Aspiration
TESE	Testicular Sperm Extraction
TGFB	Transforming Growth Factor-Beta
WHO	World Health Organization

## References

[1] Hull M.G.R., Glazener C.M.A., Kelly N.J., Conway D.I., Foster P.A., Hinton R.A., Coulson C., Lambert P.A., Watt E.M., Desai K.M. (1985). Population Study of Causes, Treatment, and Outcome of Infertility. Br. Med. J. (Clin. Res. Ed.).

[2] Boitrelle F., Shah R., Saleh R., Henkel R., Kandil H., Chung E., Vogiatzi P., Zini A., Arafa M., Agarwal A. (2021). The Sixth Edition of the WHO Manual for Human Semen Analysis: A Critical Review and SWOT Analysis. Life.

[3] Cerván-Martín M., Castilla J.A., Palomino-Morales R.J., Carmona F.D. (2020). Genetic Landscape of Nonobstructive Azoospermia and New Perspectives for the Clinic. J. Clin. Med..

[4] Wang J., Wang S., Wang M., Yang J. (2025). Analysis of Genes Implicated in Non-Obstructive Azoospermia. Steroids.

[5] Palermo G., Joris H., Devroey P., Van Steirteghem A.C. (1992). Pregnancies after Intracytoplasmic Injection of Single Spermatozoon into an Oocyte. Lancet.

[6] Devroey P., Liu J., Nagy Z., Goossens A., Tournaye H., Camus M., Van Steirteghem A., Silber S. (1995). Pregnancies after Testicular Sperm Extraction and Intracytoplasmic Sperm Injection in Non-Obstructive Azoospermia. Hum. Reprod..

[7] Practice Committee of the American Society for Reproductive Medicine (2018). Management of Nonobstructive Azoospermia: A Committee Opinion. Fertil. Steril..

[8] Esteves S.C., Miyaoka R., Orosz J.E., Agarwal A. (2013). An Update on Sperm Retrieval Techniques for Azoospermic Males. Clinics.

[9] Schlegel P.N., Li P.S. (1998). Microdissection TESE: Sperm Retrieval in Non-Obstructive Azoospermia. Hum. Reprod. Update.

[10] Khurana K.K., Sabanegh E.S. (2013). Office-Based Sperm Retrieval for Treatment of Infertility. Urol. Clin. N. Am..

[11] Vloeberghs V., Verheyen G., Haentjens P., Goossens A., Polyzos N.P., Tournaye H. (2015). How Successful Is TESE-ICSI in Couples with Non-Obstructive Azoospermia?. Hum. Reprod..

[12] Marinaro J., Goldstein M. (2022). Microsurgical Management of Male Infertility: Compelling Evidence That Collaboration with Qualified Male Reproductive Urologists Enhances Assisted Reproductive Technology (ART) Outcomes. J. Clin. Med..

[13] Deruyver Y., Vanderschueren D., Van der Aa F. (2014). Outcome of Microdissection TESE Compared with Conventional TESE in Non-obstructive Azoospermia: A Systematic Review. Andrology.

[14] Tsujimura A. (2007). Microdissection Testicular Sperm Extraction: Prediction, Outcome, and Complications. Int. J. Urol..

[15] Krausz C., Riera-Escamilla A., Moreno-Mendoza D., Holleman K., Cioppi F., Algaba F., Pybus M., Friedrich C., Wyrwoll M.J., Casamonti E. (2020). Genetic Dissection of Spermatogenic Arrest through Exome Analysis: Clinical Implications for the Management of Azoospermic Men. Genet. Med..

[16] Eliveld J., van Wely M., Meicßner A., Repping S., van der Veen F., van Pelt A.M.M. (2018). The risk of TESE-Induced Hypogonadism: A Systematic Review and Meta-Analysis. Hum. Reprod. Update.

[17] Eken A., Gulec F. (2018). Microdissection Testicular Sperm Extraction (Micro-TESE): Predictive Value of Preoperative Hormonal Levels and Pathology in Non-Obstructive Azoospermia. Kaohsiung J. Med. Sci..

[18] Kaltsas A., Stavros S., Kratiras Z., Zikopoulos A., Machairiotis N., Potiris A., Dimitriadis F., Sofikitis N., Chrisofos M., Zachariou A. (2024). Predictors of Successful Testicular Sperm Extraction: A New Era for Men with Non-Obstructive Azoospermia. Biomedicines.

[19] Klami R., Tomás C., Mankonen H., Perheentupa A. (2024). ICSI Outcome after Microdissection Testicular Sperm Extraction, Testicular Sperm Aspiration and Ejaculated Sperm. Reprod. Biol..

[20] Lacey L., Henderson I., Hassan S., Hunter H., Sajjad Y., Akhtar M.A. (2021). Can Preoperative Parameters Predict Successful Sperm Retrieval and Live Birth in Couples Undergoing Testicular Sperm Extraction and Intracytoplasmic Sperm Injection for Azoospermia?. Middle East Fertil. Soc. J..

[21] Gao S., Yang X., Xiao X., Yin S., Guan Y., Chen J., Chen Y. (2022). Outcomes and Affecting Factors for ICSI and MicroTESE Treatments in Nonobstructive Azoospermia Patients with Different Etiologies: A Retrospective Analysis. Front. Endocrinol..

[22] Kim V.N. (2005). Small RNAs: Classification, Biogenesis, and Function. Mol. Cells.

[23] Shi J., Zhou T., Chen Q. (2022). Exploring the Expanding Universe of Small RNAs. Nat. Cell Biol..

[24] Yadav R.P., Kotaja N. (2014). Small RNAs in Spermatogenesis. Mol. Cell. Endocrinol..

[25] Joshi M., Sethi S., Mehta P., Kumari A., Rajender S. (2023). Small RNAs, Spermatogenesis, and Male Infertility: A Decade of Retrospect. Reprod. Biol. Endocrinol..

[26] Chan S.Y., Wan C.W.T., Law T.Y.S., Chan D.Y.L., Fok E.K.L. (2022). The Sperm Small RNA Transcriptome: Implications beyond Reproductive Disorder. Int. J. Mol. Sci..

[27] Mukherjee A., Koli S., Reddy K.V.R. (2014). Regulatory Non-Coding Transcripts in Spermatogenesis: Shedding Light on “Dark Matter”. Andrology.

[28] Zhou G., Zhang M., Zhang J., Feng Y., Xie Z., Liu S., Zhu D., Luo Y. (2022). The Gene Regulatory Role of Non-Coding RNAs in Non-Obstructive Azoospermia. Front. Endocrinol..

[29] Shi Z., Yu M., Guo T., Sui Y., Tian Z., Ni X., Chen X., Jiang M., Jiang J., Lu Y. (2024). MicroRNAs in Spermatogenesis Dysfunction and Male Infertility: Clinical Phenotypes, Mechanisms and Potential Diagnostic Biomarkers. Front. Endocrinol..

[30] Sinaei R., Jamebozorgi K., Mirshekarpour H., Poormasoumi H., Mahdizadeh A., Akbari Z., Taghizadeh E. (2023). The Role of MiRNAs in the Diagnosis and Treatment of Male Infertility: A Review Study. Egypt. J. Med. Hum. Genet..

[31] Salas-Huetos A., James E.R., Aston K.I., Carrell D.T., Jenkins T.G., Yeste M. (2020). The Role of MiRNAs in Male Human Reproduction: A Systematic Review. Andrology.

[32] Du L., Chen W., Zhang D., Cui Y., He Z. (2024). The Functions and Mechanisms of PiRNAs in Mediating Mammalian Spermatogenesis and Their Applications in Reproductive Medicine. Cell. Mol. Life Sci..

[33] Masone M.C. (2024). PiRNA Pathway Disruption in Human Infertility. Nat. Rev. Urol..

[34] Condrat C.E., Thompson D.C., Barbu M.G., Bugnar O.L., Boboc A., Cretoiu D., Suciu N., Cretoiu S.M., Voinea S.C. (2020). MiRNAs as Biomarkers in Disease: Latest Findings Regarding Their Role in Diagnosis and Prognosis. Cells.

[35] Chen J., Han C. (2023). In Vivo Functions of MiRNAs in Mammalian Spermatogenesis. Front. Cell Dev. Biol..

[36] Lian J., Zhang X., Tian H., Liang N., Wang Y., Liang C., Li X., Sun F. (2009). Altered MicroRNA Expression in Patients with Non-Obstructive Azoospermia. Reprod. Biol. Endocrinol..

[37] Piryaei F., Mozdarani H., Sadighi Gilani M.A., Rajender S., Finelli R., Darestanifarahani M., Sarli A., Mehta P., Agarwal A. (2023). Global Analysis in Nonobstructive Azoospermic Testis Identifies MiRNAs Critical to Spermatogenesis. Andrologia.

[38] Chen X., Che D., Zhang P., Li X., Yuan Q., Liu T., Guo J., Feng T., Wu L., Liao M. (2017). Profiling of MiRNAs in Porcine Germ Cells during Spermatogenesis. Reproduction.

[39] Sree S., Radhakrishnan K., Indu S., Kumar P.G. (2014). Dramatic Changes in 67 MiRNAs during Initiation of First Wave of Spermatogenesis in Mus Musculus Testis: Global Regulatory Insights Generated by MiRNA-MRNA Network Analysis. Biol. Reprod..

[40] Abu-Halima M., Hammadeh M., Backes C., Fischer U., Leidinger P., Lubbad A.M., Keller A., Meese E. (2014). Panel of Five MicroRNAs as Potential Biomarkers for the Diagnosis and Assessment of Male Infertility. Fertil. Steril..

[41] Fontana L., Sirchia S.M., Pesenti C., Colpi G.M., Miozzo M.R. (2024). Non-Invasive Biomarkers for Sperm Retrieval in Non-Obstructive Patients: A Comprehensive Review. Front. Endocrinol..

[42] Li J., Yang F., Dong L., Chang D., Yu X. (2023). Seminal Plasma Biomarkers for Predicting Successful Sperm Retrieval in Patients with Nonobstructive Azoospermia: A Narrative Review of Human Studies. Basic Clin. Androl..

[43] Willems M., Devriendt C., Olsen C., Caljon B., Janssen T., Gies I., Vloeberghs V., Tournaye H., Van Saen D., Goossens E. (2023). Micro RNA in Semen/Urine from Non-Obstructive Azoospermia Patients as Biomarkers to Predict the Presence of Testicular Spermatozoa and Spermatogonia. Life.

[44] Chen K.Q., Wei B.H., Hao S.L., Yang W.X. (2022). The PI3K/AKT Signaling Pathway: How Does It Regulate Development of Sertoli Cells and Spermatogenic Cells?. Histol. Histopathol..

[45] Deng C.-Y., Lv M., Luo B.-H., Zhao S.-Z., Mo Z.-C., Xie Y.-J. (2021). The Role of the PI3K/AKT/MTOR Signalling Pathway in Male Reproduction. Curr. Mol. Med..

[46] Zhao Y., Yang W. (2023). The Effects of Hormone-Mediated PI3K/AKT Signaling on Spermatogenesis in Sertoli Cells. Biocell.

[47] Martello A., Mellis D., Meloni M., Howarth A., Ebner D., Caporali A., Al Haj Zen A. (2018). Phenotypic MiRNA Screen Identifies MiR-26b to Promote the Growth and Survival of Endothelial Cells. Mol. Ther. Nucleic Acids.

[48] Xing X., Guo S., Zhang G., Liu Y., Bi S., Wang X., Lu Q. (2020). MiR-26a-5p Protects against Myocardial Ischemia/Reperfusion Injury by Regulating the PTEN/PI3K/AKT Signaling Pathway. Braz. J. Med. Biol. Res..

[49] Ran M., Weng B., Cao R., Li Z., Peng F., Luo H., Gao H., Chen B. (2018). MiR-26a Inhibits Proliferation and Promotes Apoptosis in Porcine Immature Sertoli Cells by Targeting the PAK2 Gene. Reprod. Domest. Anim..

[50] Teng F., Hu F., Zhang M. (2021). MicroRNA-125a-5p Modulates the Proliferation and Apoptosis of TM4 Sertoli Cells by Targeting RAB3D and Regulating the PI3K/AKT Signaling Pathway. Mol. Hum. Reprod..

[51] Fu K., Zhang L., Liu R., Shi Q., Li X., Wang M. (2020). MiR-125 Inhibited Cervical Cancer Progression by Regulating VEGF and PI3K/AKT Signaling Pathway. World J. Surg. Oncol..

[52] Cooke P.S., Nanjappa M.K., Ko C., Prins G.S., Hess R.A. (2017). Estrogens in Male Physiology. Physiol. Rev..

[53] Carreau S., de Vienne C., Galeraud-Denis I. (2008). Aromatase and Estrogens in Man Reproduction: A Review and Latest Advances. Adv. Med. Sci..

[54] Dostalova P., Zatecka E., Dvorakova-Hortova K. (2017). Of Oestrogens and Sperm: A Review of the Roles of Oestrogens and Oestrogen Receptors in Male Reproduction. Int. J. Mol. Sci..

[55] Hess R.A. (2003). Estrogen in the Adult Male Reproductive Tract: A Review. Reprod. Biol. Endocrinol..

[56] Zhao Y., Deng C., Wang J., Xiao J., Gatalica Z., Recker R.R., Xiao G.G. (2011). Let-7 Family MiRNAs Regulate Estrogen Receptor Alpha Signaling in Estrogen Receptor Positive Breast Cancer. Breast Cancer Res. Treat..

[57] Abhari A., Zarghami N., Shahnazi V., Barzegar A., Farzadi L., Karami H., Zununi Vahed S., Nouri M., Vahed Z.S., Significance N.M. (2014). Significance of MicroRNA Targeted Estrogen Receptor in Male Fertility. Iran. J. Basic Med. Sci..

[58] Guarducci E., Nuti F., Becherini L., Rotondi M., Balercia G., Forti G., Krausz C. (2006). Estrogen Receptor Alpha Promoter Polymorphism: Stronger Estrogen Action Is Coupled with Lower Sperm Count. Hum. Reprod..

[59] Christian M., W-F Lam E., SC Wilson M., J Brosens J. (2011). FOXO Transcription Factors and Their Role in Disorders of the Female Reproductive Tract. Curr. Drug Targets.

[60] Goertz M.J., Wu Z., Gallardo T.D., Hamra F.K., Castrillon D.H. (2011). Foxo1 Is Required in Mouse Spermatogonial Stem Cells for Their Maintenance and the Initiation of Spermatogenesis. J. Clin. Investig..

[61] Duwe L., Munoz-Garrido P., Lewinska M., Lafuente-Barquero J., Satriano L., Høgdall D., Taranta A., Nielsen B.S., Ghazal A., Matter M.S. (2023). MicroRNA-27a-3p Targets FoxO Signalling to Induce Tumour-like Phenotypes in Bile Duct Cells. J. Hepatol..

[62] Young J.C., Wakitani S., Loveland K.L. (2015). TGF-β Superfamily Signaling in Testis Formation and Early Male Germline Development. Semin. Cell Dev. Biol..

[63] Moreno S.G., Attali M., Allemand I., Messiaen S., Fouchet P., Coffigny H., Romeo P.H., Habert R. (2010). TGFβ Signaling in Male Germ Cells Regulates Gonocyte Quiescence and Fertility in Mice. Dev. Biol..

[64] Loveland K.L., Hogarth C., Mendis S., Efthymiadis A., Ly J., Itman C., Meachem S., Brown C.W., Jans D.A. (2005). Drivers of Germ Cell Maturation. Ann. N. Y. Acad. Sci..

[65] Liu W., Du L., Li J., He Y., Tang M. (2024). Microenvironment of Spermatogonial Stem Cells: A Key Factor in the Regulation of Spermatogenesis. Stem Cell Res. Ther..

[66] Abé K., Eto K., Abé S.I. (2008). Epidermal Growth Factor Mediates Spermatogonial Proliferation in Newt Testis. Reprod. Biol. Endocrinol..

[67] Aminmalek M., Mashayekhi F., Salehi Z. (2021). Epidermal Growth Factor +61A/G (RS4444903) Promoter Polymorphism and Serum Levels Are Linked to Idiopathic Male Infertility. Br. J. Biomed. Sci..

[68] Saucedo L., Buffa G.N., Rosso M., Guillardoy T., Góngora A., Munuce M.J., Vazquez-Levin M.H., Marín-Briggiler C. (2015). Fibroblast Growth Factor Receptors (FGFRs) in Human Sperm: Expression, Functionality and Involvement in Motility Regulation. PLoS ONE.

[69] Cotton L.M., O’Bryan M.K., Hinton B.T. (2008). Cellular Signaling by Fibroblast Growth Factors (FGFs) and Their Receptors (FGFRs) in Male Reproduction. Endocr. Rev..

[70] Metallinou C., Staneloudi C., Nikolettos K., Asimakopoulos B. (2024). NGF, EPO, and IGF-1 in the Male Reproductive System. J. Clin. Med..

[71] Kierszenbaum A.L. (2006). Tyrosine Protein Kinases and Spermatogenesis: Truncation Matters. Mol. Reprod. Dev..

[72] Chen Y., Wang H., Qi N., Wu H., Xiong W., Ma J., Lu Q., Han D. (2009). Functions of TAM RTKs in Regulating Spermatogenesis and Male Fertility in Mice. Reproduction.

[73] Xiong W., Chen Y., Wang H., Wang H., Wu H., Lu Q., Han D. (2008). Gas6 and the Tyro 3 Receptor Tyrosine Kinase Subfamily Regulate the Phagocytic Function of Sertoli Cells. Reproduction.

[74] Chen Y., Wei H.-F., Song C.-W., Zhu C.-C. (2022). Discoidin Domain Receptor 2 Expression Increases Phagocytotic Capacity in Sertoli Cells of Sertoli Cell-Only Syndrome Testes. Am. J. Transl. Res..

[75] Kuang H., Zhang C., Zhang W., Cai H., Yang L., Yuan N., Yuan Y., Yang Y., Zuo C., Zhong F. (2022). Electroacupuncture Improves Intestinal Motility through Exosomal MiR-34c-5p Targeting SCF/c-Kit Signaling Pathway in Slow Transit Constipation Model Rats. Evid.-Based Complement. Alternat. Med..

[76] Feng H.L., Sandlow J.I., Sparks A.E.T., Sandra A., Zheng L.J. (1999). Decreased Expression of the C-Kit Receptor is Associated with Increased Apoptosis in Subfertile Human Testes. Fertil. Steril..

[77] Qin W.Y., Feng S.C., Sun Y.Q., Jiang G.Q. (2020). MiR-96-5p Promotes Breast Cancer Migration by Activating MEK/ERK Signaling. J. Gene Med..

[78] Zolfaghari N., Soheili Z.S., Samiei S., Latifi-Navid H., Hafezi-Moghadam A., Ahmadieh H., Rezaei-Kanavi M. (2023). MicroRNA-96 Targets the INS/AKT/GLUT4 Signaling Axis: Association with and Effect on Diabetic Retinopathy. Heliyon.

[79] Lackey B.R., Gray S.L. (2015). Second Messengers, Steroids and Signaling Cascades: Crosstalk in Sperm Development and Function. Gen. Comp. Endocrinol..

[80] Loveland K.L., Klein B., Pueschl D., Indumathy S., Bergmann M., Loveland B.E., Hedger M.P., Schuppe H.C. (2017). Cytokines in Male Fertility and Reproductive Pathologies: Immunoregulation and Beyond. Front. Endocrinol..

[81] Syriou V., Papanikolaou D., Kozyraki A., Goulis D.G. (2018). Cytokines and Male Infertility. Eur. Cytokine Netw..

[82] Dutta S., Sengupta P., Slama P., Roychoudhury S. (2021). Oxidative Stress, Testicular Inflammatory Pathways, and Male Reproduction. Int. J. Mol. Sci..

[83] Hu J., Huang S., Liu X., Zhang Y., Wei S., Hu X. (2022). MiR-155: An Important Role in Inflammation Response. J. Immunol. Res..

[84] Xu J., He C., Fang Y.W., Hu Z.Y., Peng M.L., Chen Y.Y., Su Y.F., Liu C.Y., Zhang H.P., Zhao K. (2022). Testicular Exosomes Disturb the Immunosuppressive Phenotype of Testicular Macrophages Mediated by MiR-155-5p in Uropathogenic Escherichia Coli-Induced Orchitis. Asian J. Androl..

[85] Doherty C.M., Tarchala S.M., Radwanska E., de Jonge C.J. (1995). Characterization of Two Second Messenger Pathways and Their Interactions in Eliciting the Human Sperm Acrosome Reaction. J. Androl..

[86] Xu K., Chen C., Wu Y., Wu M., Lin L. (2021). Advances in MiR-132-Based Biomarker and Therapeutic Potential in the Cardiovascular System. Front. Pharmacol..

[87] Stewart T.A., Davis F.M. (2019). An Element for Development: Calcium Signaling in Mammalian Reproduction and Development. Biochim. Biophys. Acta (BBA)-Mol. Cell Res..

[88] Finkelstein M., Etkovitz N., Breitbart H. (2020). Ca^2+^ Signaling in Mammalian Spermatozoa. Mol. Cell. Endocrinol..

[89] Guido C., Perrotta I., Panza S., Middea E., Avena P., Santoro M., Marsico S., Imbrogno P., Andò S., Aquila S. (2011). Human Sperm Physiology: Estrogen Receptor Alpha (ERα) and Estrogen Receptor Beta (ERβ) Influence Sperm Metabolism and May Be Involved in the Pathophysiology of Varicocele-Associated Male Infertility. J. Cell. Physiol..

[90] Hess R.A., Cooke P.S. (2018). Estrogen in the Male: A Historical Perspective. Biol. Reprod..

[91] Ded L., Dostalova P., Dorosh A., Dvorakova-Hortova K., Peknicova J. (2010). Effect of Estrogens on Boar Sperm Capacitation in Vitro. Reprod. Biol. Endocrinol..

[92] Martinot E., Sedes L., Baptissart M., Holota H., Angelique D.H., Beaudoin C., Volle D.H. (2018). Nuclear Receptor Networks in Male Fertility. Endocr. Abstr..

[93] Monrose M., Thirouard L., Garcia M., Holota H., De Haze A., Caira F., Beaudoin C., Volle D.H. (2021). New Perspectives on PPAR, VDR and FXRα as New Actors in Testicular Pathophysiology. Mol. Aspects Med..

[94] O’Hara L., Smith L.B. (2015). Androgen Receptor Roles in Spermatogenesis and Infertility. Best Pract. Res. Clin. Endocrinol. Metab..

[95] Dohle G.R., Smit M., Weber R.F.A. (2003). Androgens and Male Fertility. World J. Urol..

[96] Zhao Y., Deng S., Li C., Cao J., Wu A., Chen M., Ma X., Wu S., Lian Z. (2024). The Role of Retinoic Acid in Spermatogenesis and Its Application in Male Reproduction. Cells.

[97] Pakpahan C., Margiana R., Pangestu M. (2022). A Systematic Review of Retinoic Acid in the Journey of Spermatogonium to Spermatozoa: From Basic to Clinical Application. F1000Research.

[98] Villani M.T., Morini D., Spaggiari G., Falbo A.I., Melli B., La Sala G.B., Romeo M., Simoni M., Aguzzoli L., Santi D. (2021). Are Sperm Parameters Able to Predict the Success of Assisted Reproductive Technology? A Retrospective Analysis of over 22,000 Assisted Reproductive Technology Cycles. Andrology.

[99] Conflitti A.C., Cicolani G., Buonacquisto A., Pallotti F., Faja F., Bianchini S., Blaconà G., Bruno S.M., Linari A., Lucarelli M. (2023). Sperm DNA Fragmentation and Sperm-Borne MiRNAs: Molecular Biomarkers of Embryo Development?. Int. J. Mol. Sci..

[100] Abu-Halima M., Backes C., Leidinger P., Keller A., Lubbad A.M., Hammadeh M., Meese E. (2014). MicroRNA Expression Profiles in Human Testicular Tissues of Infertile Men with Different Histopathologic Patterns. Fertil. Steril..

[101] Dorostghoal M., Galehdari H., Hemadi M., Davoodi E. (2022). Sperm MiR-34c-5p Transcript Content and Its Association with Sperm Parameters in Unexplained Infertile Men. Reprod. Sci..

[102] Finocchi F., Pelloni M., Balercia G., Pallotti F., Radicioni A.F., Lenzi A., Lombardo F., Paoli D. (2020). Seminal Plasma MiRNAs in Klinefelter Syndrome and in Obstructive and Non-Obstructive Azoospermia. Mol. Biol. Rep..

[103] Momeni A., Najafipour R., Hamta A., Jahani S., Moghbelinejad S. (2020). Expression and Methylation Pattern of Hsa-MiR-34 Family in Sperm Samples of Infertile Men. Reprod. Sci..

[104] Fang N., Cao C., Wen Y., Wang X., Yuan S., Huang X. (2019). MicroRNA Profile Comparison of Testicular Tissues Derived from Successful and Unsuccessful Microdissection Testicular Sperm Extraction Retrieval in Non-Obstructive Azoospermia Patients. Reprod. Fertil. Dev..

[105] Cui L., Fang L., Shi B., Qiu S., Ye Y. (2015). Spermatozoa Micro Ribonucleic Acid–34c Level Is Correlated with Intracytoplasmic Sperm Injection Outcomes. Fertil. Steril..

[106] Pantos K., Grigoriadis S., Tomara P., Louka I., Maziotis E., Pantou A., Nitsos N., Vaxevanoglou T., Kokkali G., Agarwal A. (2021). Investigating the Role of the MicroRNA-34/449 Family in Male Infertility: A Critical Analysis and Review of the Literature. Front. Endocrinol..

[107] Coultas L., Bouillet P., Loveland K.L., Meachem S., Perlman H., Adams J.M., Strasser A. (2005). Concomitant Loss of Proapoptotic BH3-Only Bcl-2 Antagonists Bik and Bim Arrests Spermatogenesis. EMBO J..

[108] Zhang L., Tang J., Haines C.J., Feng H.L., Lai L., Teng X., Han Y. (2011). C-Kit and Its Related Genes in Spermatogonial Differentiation. Spermatogenesis.

[109] Hirsch M.N., Pallotti F., Faja F., Buonacquisto A., Cicolani G., Conflitti A.C., Di Chiano S., Lenzi A., Lombardo F., Paoli D. (2023). Bladder Neck Obstruction: Experience and Management in a Sperm Bank. Life.

[110] Mekhaimar A., Goble M., Brunckhorst O., Alnajjar H.M., Ralph D., Muneer A., Ahmed K. (2020). A Systematic Review of Transurethral Resection of Ejaculatory Ducts for the Management of Ejaculatory Duct Obstruction. Turk. J. Urol..

[111] Achermann A.P.P., Esteves S.C. (2020). Diagnosis and Management of Infertility Due to Ejaculatory Duct Obstruction: Summary Evidence. Int. Braz. J. Urol. Off. J. Braz. Soc. Urol..

[112] Jarvi K., Lo K., Grober E., Mak V., Fischer A., Grantmyre J., Zini A., Chan P., Patry G., Chow V. (2015). The Workup and Management of Azoospermic Males. Can. Urol. Assoc. J..

[113] Du Plessis S.S., Agarwal A., Sabanegh E.S. (2014). Male Infertility: A Complete Guide to Lifestyle and Environmental Factors.

[114] Chen S., An G., Wang H., Wu X., Ping P., Hu L., Chen Y., Fan J., Cheng C.Y., Sun F. (2022). Human Obstructive (Postvasectomy) and Nonobstructive Azoospermia–Insights from ScRNA-Seq and Transcriptome Analysis. Genes Dis..

[115] Oraiopoulou C., Vorniotaki A., Taki E., Papatheodorou A., Christoforidis N., Chatziparasidou A. (2021). The Impact of Fresh and Frozen Testicular Tissue Quality on Embryological and Clinical Outcomes. Andrologia.

[116] Bolger A.M., Lohse M., Usadel B. (2014). Trimmomatic: A Flexible Trimmer for Illumina Sequence Data. Bioinformatics.

[117] Langmead B., Salzberg S.L. (2012). Fast Gapped-Read Alignment with Bowtie 2. Nat. Methods.

[118] Langmead B., Wilks C., Antonescu V., Charles R. (2019). Scaling Read Aligners to Hundreds of Threads on General-Purpose Processors. Bioinformatics.

[119] Kozomara A., Birgaoanu M., Griffiths-Jones S. (2019). MiRBase: From MicroRNA Sequences to Function. Nucleic Acids Res..

[120] Friedländer M.R., MacKowiak S.D., Li N., Chen W., Rajewsky N. (2012). MiRDeep2 Accurately Identifies Known and Hundreds of Novel MicroRNA Genes in Seven Animal Clades. Nucleic Acids Res..

[121] Aparicio-Puerta E., Lebrón R., Rueda A., Gómez-Martín C., Giannoukakos S., Jaspez D., Medina J.M., Zubkovic A., Jurak I., Fromm B. (2019). SRNAbench and SRNAtoolbox 2019: Intuitive Fast Small RNA Profiling and Differential Expression. Nucleic Acids Res..

[122] Ontiveros-Palacios N., Cooke E., Nawrocki E.P., Triebel S., Marz M., Rivas E., Griffiths-Jones S., Petrov A.I., Bateman A., Sweeney B. (2013). Rfam 15: RNA Families Database in 2025. Nucleic Acids Res..

[123] Wen M., Shen Y., Shi S., Tang T. (2012). MiREvo: An Integrative MicroRNA Evolutionary Analysis Platform for Next-Generation Sequencing Experiments. BMC Bioinform..

[124] Love M.I., Huber W., Anders S. (2014). Moderated Estimation of Fold Change and Dispersion for RNA-Seq Data with DESeq2. Genome Biol..

[125] Storey J.D. (2003). The Positive False Discovery Rate: A Bayesian Interpretation and the q-Value. Ann. Stat..

[126] Betel D., Wilson M., Gabow A., Marks D.S., Sander C. (2008). The MicroRNA.Org Resource: Targets and Expression. Nucleic Acids Res..

[127] Ashburner M., Ball C.A., Blake J.A., Botstein D., Butler H., Cherry J.M., Davis A.P., Dolinski K., Dwight S.S., Eppig J.T. (2000). Gene Ontology: Tool for the Unification of Biology. Nat. Genet..

[128] Consortium T.G.O., Aleksander S.A., Balhoff J., Carbon S., Cherry J.M., Drabkin H.J., Ebert D., Feuermann M., Gaudet P., Harris N.L. (2023). The Gene Ontology Knowledgebase in 2023. Genetics.

[129] Kanehisa M., Sato Y., Kawashima M., Furumichi M., Tanabe M. (2016). KEGG as a Reference Resource for Gene and Protein Annotation. Nucleic Acids Res..

[130] Kanehisa M., Furumichi M., Sato Y., Kawashima M., Ishiguro-Watanabe M. (2023). KEGG for Taxonomy-Based Analysis of Pathways and Genomes. Nucleic Acids Res..

[131] Wu J., Mao X., Cai T., Luo J., Wei L. (2006). KOBAS Server: A Web-Based Platform for Automated Annotation and Pathway Identification. Nucleic Acids Res..

