# Supplementary material for: The Small RNA Landscape in Azoospermia: Implications for Male Infertility and Sperm Retrieval—A Preliminary Study
Source: Int J Mol Sci. 2025 Apr 9;26(8):3537. doi: 10.3390/ijms26083537 (PMC12027063; doi:10.3390/ijms26083537)
Supplement: Supplementary file 1 [file ijms-26-03537-s001.zip › ijms-3509610-supplementary.pdf]

Patient Questionnaire

Date:

Occupation:

Nationality:

Weight:

Height:

Previous Semen Analysis Tests:

Have you ever had a positive semen culture?

If yes, specify the microorganism:

Are you trying to conceive?

Do you have children or have you ever resulted in a confirmed pregnancy?

Do you have siblings?

Mother's age at conception:

Was she a smoker?

Father's age at conception:

Was he a smoker?

Are you a smoker?

If yes, how much do you smoke?

Have you quit smoking? If so, when?

Have you ever used drugs?

If yes, when?

Alcohol consumption per week:

Are you taking any medication?

If yes, specify the medication and the condition it is used for:

Have you had a fever in the last three months?

Have you had a viral infection in the last month?

Have you had any surgery in the genital area?

If YES: When? What type?

Have you recently visited hot baths, saunas, or spas?

Are you exposed to chemicals in your everyday life?

Do you frequently use a laptop resting on your body?

Do you take any dietary supplements, herbal remedies, or vitamins?

Do you follow homeopathy or any other alternative treatment?

What is your lifestyle:

How would you describe your daily diet?

Do you exercise?

Additional Comments:
